# Synthetic receptor platform to identify loss-of-function single nucleotide variants and designed mutants in the death receptor Fas/CD95

**DOI:** 10.1016/j.jbc.2023.104989

**Published:** 2023-06-29

**Authors:** Anna Rita Minafra, Puyan Rafii, Sofie Mossner, Farhad Bazgir, Doreen M. Floss, Jens M. Moll, Jürgen Scheller

**Affiliations:** 1 Institute of Biochemistry and Molecular Biology II, Medical Faculty, Heinrich-Heine-University, Düsseldorf, Germany; 2 PROvendis GmbH, Muelheim an der Ruhr, Germany

**Keywords:** cytokine, CD95 (APO-1/Fas, single-nucleotide polymorphism (SNP), synthetic biology, single-domain antibody (sdAb, nanobody), signal transduction

## Abstract

Synthetic biology has emerged as a useful technology for studying cytokine signal transduction. Recently, we described fully synthetic cytokine receptors to phenocopy trimeric receptors such as the death receptor Fas/CD95. Using a nanobody as an extracellular-binding domain for mCherry fused to the natural receptor’s transmembrane and intracellular domain, trimeric mCherry ligands were able to induce cell death. Among the 17,889 single nucleotide variants in the SNP database for Fas, 337 represent missense mutations that functionally remained largely uncharacterized. Here, we developed a workflow for the Fas synthetic cytokine receptor system to functionally characterize missense SNPs within the transmembrane and intracellular domain of Fas. To validate our system, we selected five functionally assigned loss-of-function (LOF) polymorphisms and included 15 additional unassigned SNPs. Moreover, based on structural data, 15 gain-of-function or LOF candidate mutations were additionally selected. All 35 nucleotide variants were functionally investigated through cellular proliferation, apoptosis and caspases 3 and 7 cleavage assays. Collectively, our results showed that 30 variants resulted in partial or complete LOF, while five lead to a gain-of-function. In conclusion, we demonstrated that synthetic cytokine receptors are a suitable tool for functional SNPs/mutations characterization in a structured workflow.

Immunoregulatory cytokines, including tumor necrosis factor (TNF) and FasL, control immune-related events and are critically involved in pathophysiological processes such as autoimmunity and cancer (1). Fas/CD95 is a death receptor belonging to the TNF superfamily, characterized by a conserved intracellular death domain (DD) (2). Upon trimerized FasL binding, conformational changes in the intracellular DD result in the binding of the adapter molecule fas-associated death domain (FADD) and activation of procaspase 8 *via* the death-inducing–signaling complex (3). Following autocatalytic cleavage, active caspase 8 promotes downstream signaling, including effector caspases 3 and 7 that lead to final apoptosis (4).

Synthetic biology has become an alternative option to analyze cytokine signal transduction as well as for the development of personalized therapies (5). Recently, we generated fully synthetic cytokine receptors to phenocopy prototypical trimeric receptors for TNF and FasL (6). In our synthetic cytokine receptors for Fas (Fas-SyCyR), we use nanobodies as extracellular ligand-binding domains (7, 8), fused to the transmembrane and intracellular domain of the receptor of interest. The nanobodies serve as biosensors for homomeric or heteromeric ligands, for example, fusion proteins of GFP and mCherry (9). Activation of the endogenous signaling pathway is followed by binding of the synthetic ligand to the synthetic receptor (10, 11).

Nonsynonymous SNPs in Fas eventually cause defects in lymphocyte apoptosis leading to autoimmune diseases and cancer, such as the development of autoimmune lymphoproliferative syndrome (ALPS) (12) and squamous cell carcinoma (SCC) (13). In general, SNPs might influence gene expression, protein folding, stability, localization, or function (14, 15). Apropos of Fas, functional analysis of most nonsynonymous, coding SNPs to understand the molecular mechanism(s) that could cause defective apoptosis and/or a link to disease has not been investigated or found for the vast majority of nonsynonymous SNPs, most likely because they were found in more random-like genome sequencing approaches and not by an underlying disease-driven sequencing strategy. Therefore, combining systematic bioinformatics and experimental approaches is needed to manage the expanding SNP landscape. Functionally relevant SNPs can be predicted by in-depth structure-guided analysis (16) or by basic online data processing tools such as Provean (17). Moreover, recent developments in artificial intelligence enable the *in silico* prediction of structures of proteins and protein complexes (18). These deep learning algorithms including RoseTTAFold (19) and AlphaFold (20) will aid the structural understanding of amino acid exchanges either in the exploration phase to screen for functionally relevant SNPs or in the postlaboratory phase to understand the biochemically approved gain-of-function (GOF) or loss-of-function (LOF) SNPs. However, *in silico* characterization has to be experimentally validated, at least for GOF and LOF mutants.

Here, we developed an experimental workflow for the Fas-SyCyR system to functionally characterize SNPs and structure-predicted mutations within the transmembrane and intracellular (death) domain of Fas/CD95. We used the SNP database (dbSNP) and clinical variants (ClinVar) database, compilations of all known polymorphisms and polymorphisms with a clinical correlation. Provean and structure-guided analysis were used for the preselection of candidate GOF and LOF mutations. Mutations were introduced into Fas-SyCyR, followed by a functional quantitative characterization including cellular proliferation, apoptosis, and effector caspases 3 and 7 cleavage assays.

In summary, our results showed that among the 35 functionally characterized mutants, 22 were strong LOF, eight were mild LOF, and five led to a mild GOF. We comprehensively demonstrated that the Fas-SyCyR system is a valid tool to functionally and systematically characterize LOF and GOF variants.

## Results

### Selection of SNPs in the transmembrane and intracellular domain of Fas/CD95

Among the 17,889 SNPs found in the Fas gene, we surveyed the listed 337 nonsynonymous missense SNPs of the transmembrane and intracellular domain, of which 39 were listed in ClinVar. From the 337 missense SNPs, 23 were reported in peer-reviewed publications, 19 had a direct disease association but only 13 were experimentally validated (21, 22).

**Figure 1:**
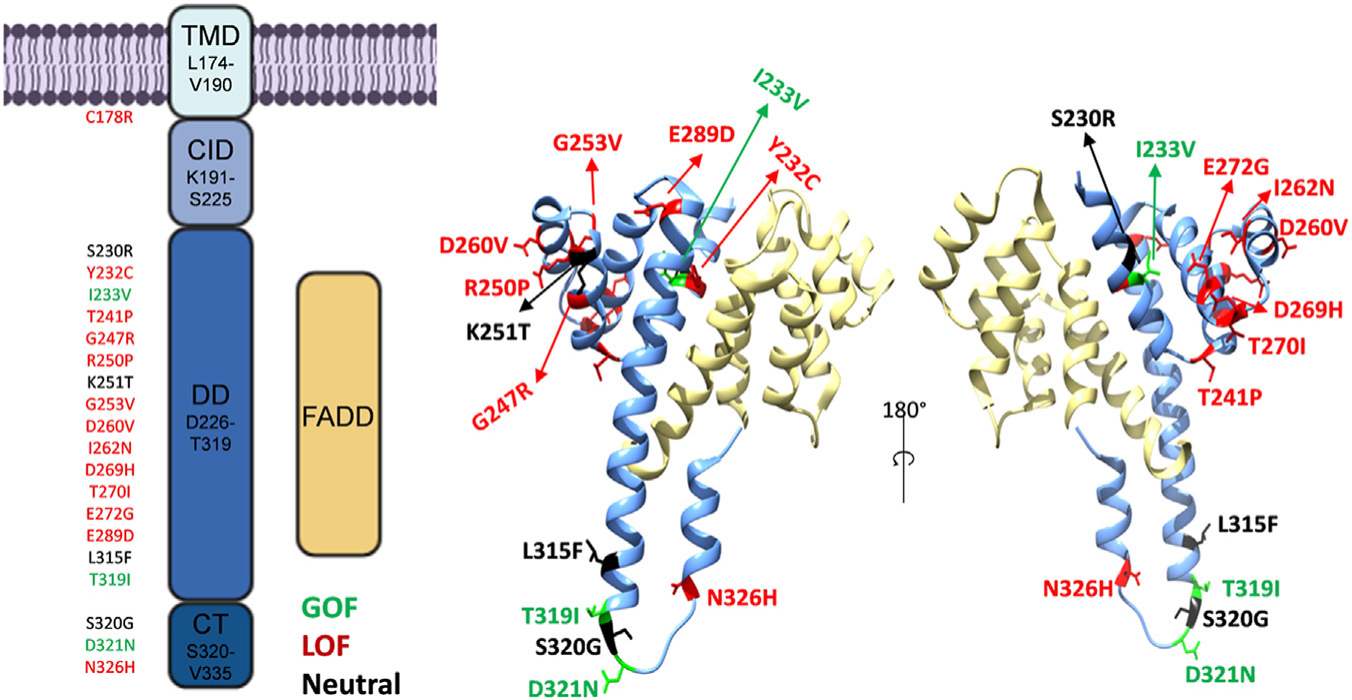
**Schematic representation of Fas synthetic receptor and SNPs localization.** Graphic representation of transmembrane domain (TMD) and intracellular domain (ICD) of Fas synthetic receptor, including calcium-inducing domain (CID), death domain (DD) interacting with FADD, and COOH-terminal region (CT) (3EZQ). All the selected SNPs’ location is highlighted in the Fas TMD, DD, and CT. The final activity is indicated by the following color legend: *red*, LOF; *green*, GOF; and *black*, neutral. GOF, gain-of-function; LOF, loss-of-function.

Nonsynonymous SNPs reported in ClinVar have a high probability of being LOF, whereas SNPs listed only in dbSNP might have no effect on protein function. To identify further nonsynonymous SNPs with potential LOF characteristics among the ones listed in dbSNP, we analyzed all nonsynonymous dbSNPs with Provean. We then selected ten previously uncharacterized nonsynonymous SNPs from ClinVar and five Provean predicted SNPs from dbSNP (ClinVar: C178R, G253V, I262N, E272G, E289D, L315F, T319I, S320G, D321N, N326H; Provean: S230R, I233V, G247R, K251T, D269H). Moreover, we included the five previously characterized nonsynonymous LOF SNPs Y232C, T241P, R250P, D260V, and T270I located in the intracellular DD, which were reported to be associated with ALPS and SCC (23, 24, 25, 26, 27, 28, 29, 30). Figure 1 shows the structural localization of the selected SNPs in the Fas/FADD complex structure.

### LOF SNPs in Fas-SyCyR failed to inhibit the proliferation of Ba/F3-gp130 cells

**Figure 2:**
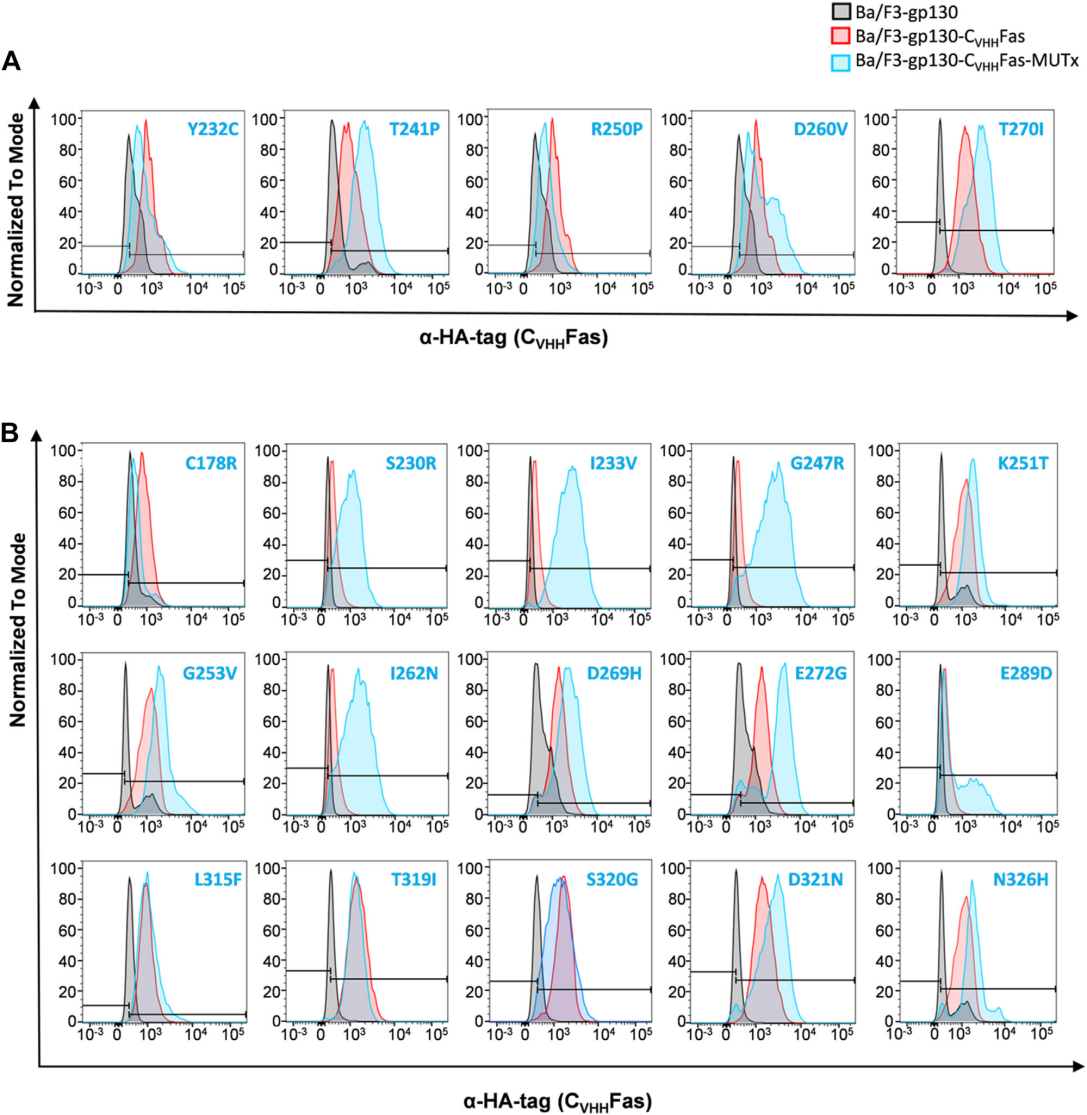
**Test expression of WT and mutated C**_**VHH**_**Fas.***A*, expression of previously published SNPs (Y232C, T241P, R250P, D260V, T270I) by specific detection of surface α-HA-tag through flow cytometry. *Black color*: Ba/F3-gp130 cells; *red color*: Ba/F3-gp130-C_VHH_Fas cells; *blue color*: Ba/F3-gp130-C_VHH_Fas with indicated variant. *B*, test expression of ClinVar reported and Provean predicted SNPs (C178R, S230R, I233V, G247R, K251T, G253V, I262N, D269H, E272G, E289D, L315F, T319I, S320G, D321N, and N326H). *Black color*: Ba/F3-gp130 cells; *red color*: Ba/F3-gp130-C_VHH_Fas cells; *blue color*: Ba/F3-gp130-C_VHH_Fas with indicated variant. ClinVar, clinical variant.

The 20 selected nonsynonymous missense SNPs were introduced into the complementary DNA (cDNA) coding for the synthetic Fas-SyCyR (C_VHH_Fas) receptor and introduced into Ba/F3-gp130 cells. Cell surface expression of mutant C_VHH_Fas (with N-terminal human influenza hemagglutinin [HA]-tag) was verified by flow cytometry using HA antibodies (Fig. 2, *A* and *B*).

**Figure 3:**
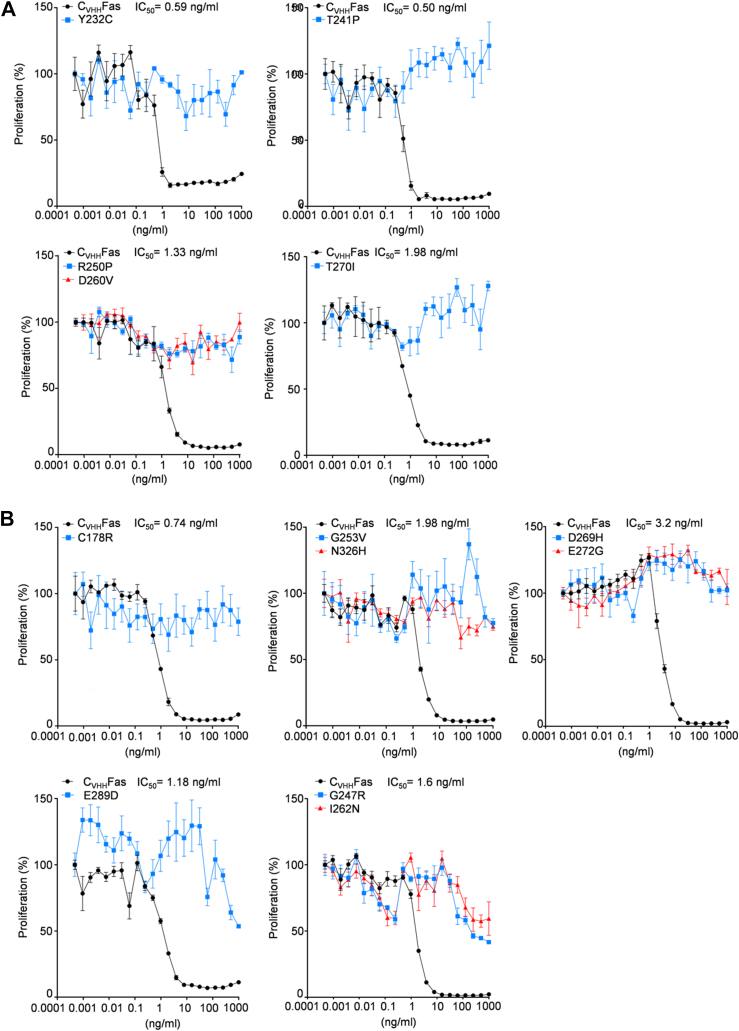
**LOF Fas variants failed to inhibit HIL-6–induced cellular proliferation.***A*, Ba/F3-gp130 cells expressing previously published, LOF SNPs (Y232C, T241P, R250P, D260V, T270I) in C_VHH_Fas were treated with 20 ng/ml HIL-6 and increasing concentration of 3C (from 1 × 10^−4^ to 1 × 10^3^ ng/ml) for 72 h. *Black color*: Ba/F3-gp130-C_VHH_Fas cells; *blue color*: Ba/F3-gp130-C_VHH_Fas with indicated variant. *B*, Ba/F3-gp130 cells expressing up to date uncharacterized, LOF SNPs (C178R, G247R, G253V, I262N, D269H, E272G, E289D, and N326H). Cells were treated as described above. *Black color*: Ba/F3-gp130-C_VHH_Fas cells; *blue* or *red colors*: Ba/F3-gp130-C_VHH_Fas with indicated variant. Error bars indicate ± SEM. HIL-6, Hyper-IL-6; LOF, loss-of-function.

Apoptosis of Ba/F3-gp130-C_VHH_Fas cells was induced upon addition of trimeric mCherry (3C) fused to an Fc part of an IgG1 antibody (6). The IC_50_ of the synthetic FasL ligand was determined to be in the range of 0.5 and 3.2 ng/ml, demonstrating that low quantitative doses were sufficient to efficiently prevent Hyper-IL-6 (HIL-6)–induced proliferation of Ba/F3-gp130 cells expressing synthetic Fas as control (Fig. 3, *A* and *B*). HIL-6 is a fusion protein of IL-6 and the soluble IL-6R, which specifically activates gp130 receptor signal transduction and proliferation of Ba/F3-gp130 cells (6).

As expected, the five previously characterized LOF SNPs (Y232C, T241P, R250P, D260V, T270I) failed to inhibit HIL-6–induced proliferation (Fig. 3*A*). Among the 15 additional candidates, eight variants were also unable to inhibit HIL-6–induced cellular proliferation, thereof were six SNPs from ClinVar (C178R, G253V, I262N, E272G, E289D, N326H) and two SNPs predicted by Provean (G247R and D269H) (Fig. 3*B*).

**Figure 4:**
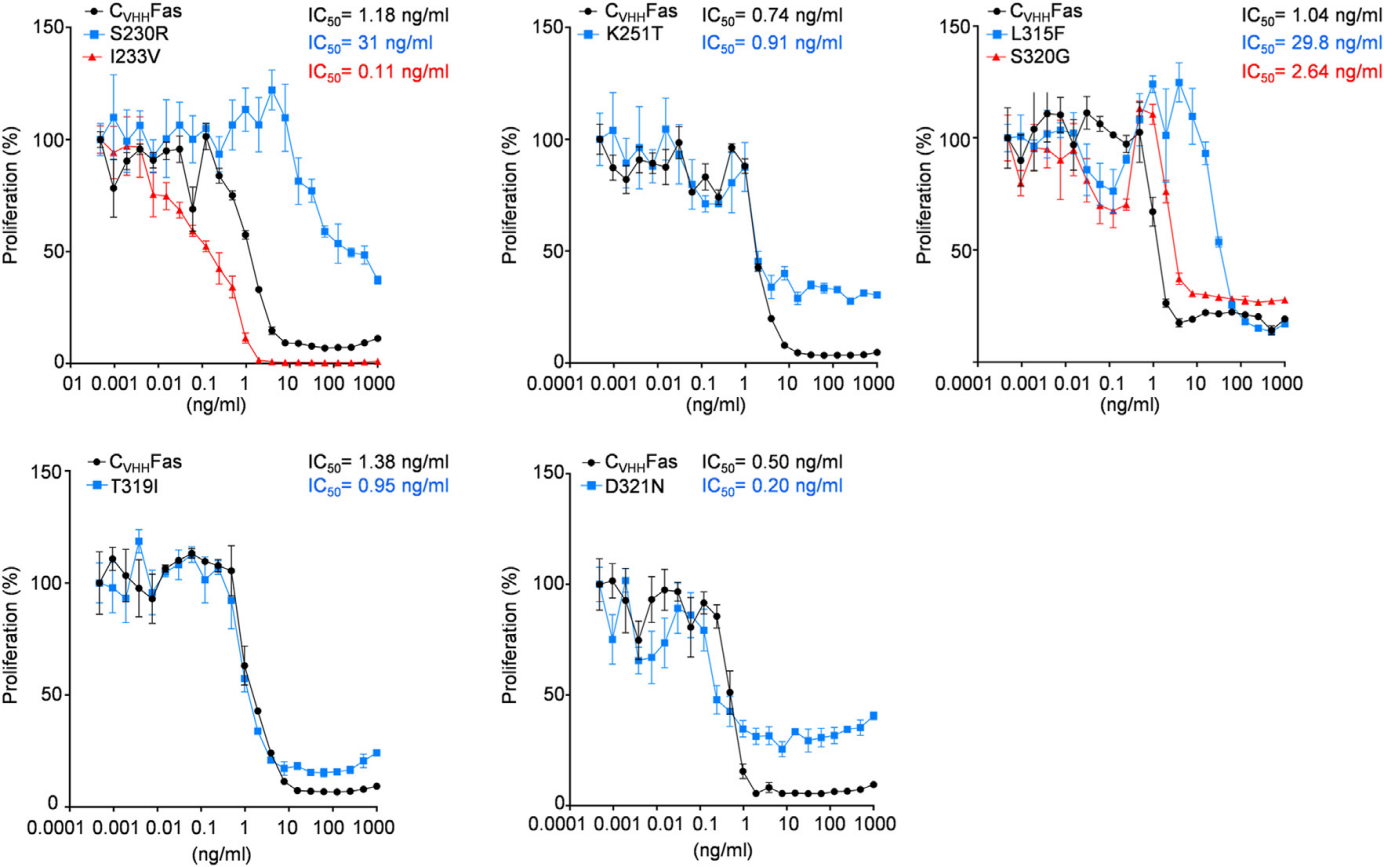
**Active SNPs are still able to inhibit HIL-6–induced cellular proliferation upon synthetic ligand stimulation.** Ba/F3-gp130 cells expressing previously uncharacterized, active SNPs (S230R, I233V, K251T, L315F, T319I, S320G, and D321N) in C_VHH_Fas were treated with 20 ng/ml HIL-6 and increasing concentration of 3C (from 1 × 10^−4^ to 1 × 10^3^ ng/ml) for 72 h. *Black color*: Ba/F3-gp130-C_VHH_Fas cells; *blue* or *red color*: Ba/F3-gp130-C_VHH_Fas with indicated variant. Error bars indicate ± SEM. HIL-6, Hyper-IL-6.

Contrary, the remaining four SNPs from ClinVar (L315F, T319I, S320G, D321N) and three mutants from the dbSNP/Provean prediction (S230R, I233V, K251T) were biologically active. The two variants S230R, L315F showed, however, a reduced IC_50_ of 31 ng/ml and 29.8 ng/ml, respectively. The remaining SNPs K251T, T319I, S320G did not change the biological activity of Fas (IC_50_ of 0.91 ng/ml, 0.95 ng/ml, and 2.64 ng/ml, respectively). Of note, the previously uncharacterized SNPs I233V and D321N were mild GOF mutations with an IC_50_ of 0.11 ng/ml and 0.20 ng/ml, respectively (Fig. 4).

Here, cellular proliferation was used to initially estimate the consequences for the biological activity of the Fas SNP mutations. In summary, 13 out of 20 SNPs are complete LOFs (Y232C, T241P, R250P, D260V, T270I, C178R, G253V, I262N, E272G, E289D, N326H, G247R, and D269H), two have mild LOFs (S230R, L315F), three have no effect (K251T, T319I, and S320G), and two are GOFs (I233V, D321N).

### Structure-predicted mutations within the DD of Fas

**Figure 5:**
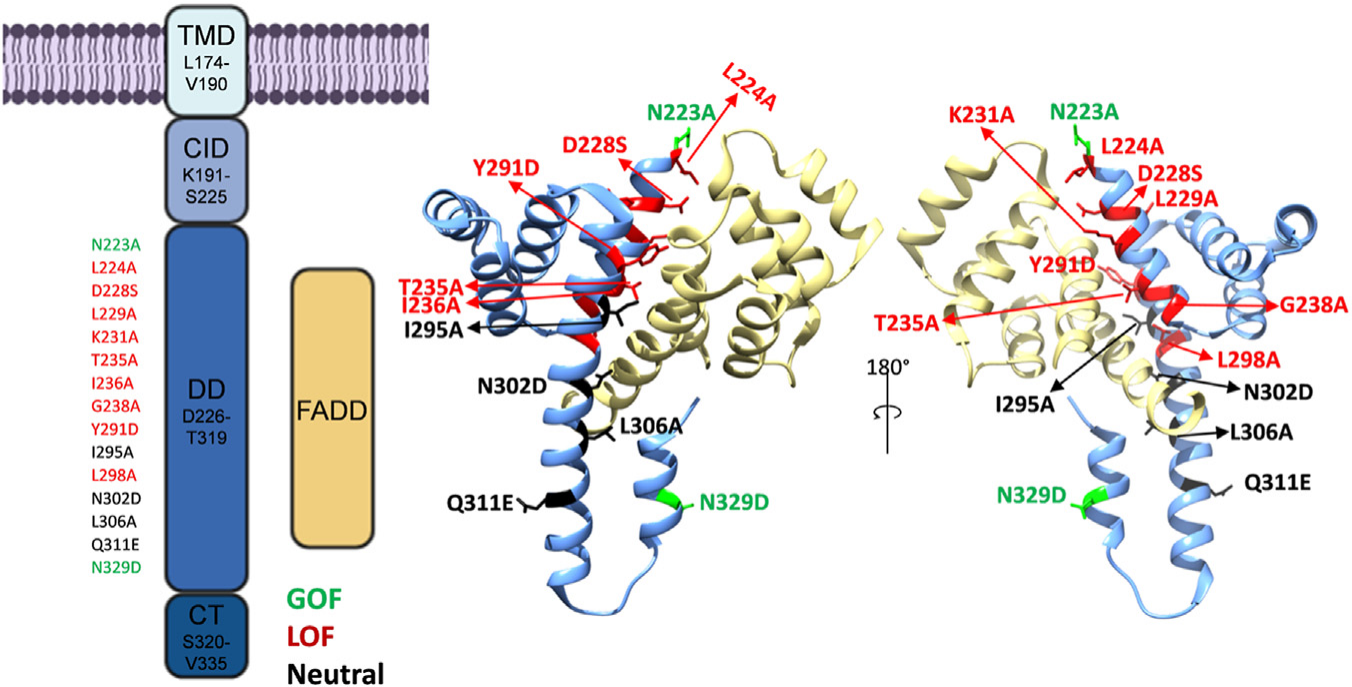
**Schematic representation of Fas synthetic receptor and structure-based mutations localization.** Fas synthetic receptor's TMD and ICD shown in interaction with FADD (3EZQ). The locations of all the chosen structure-based mutations are highlighted in the DD structure. The following color legend designates the final activity: *red*, LOF, *green*, GOF, and *black*, neutral. GOF, gain-of-function; LOF, loss-of-function.

Using structure-based prediction, we selected 15 LOF and GOF candidates. All 15 missense mutations were located in the DD and resulted in substitution into an alanine (ten alanine variants) or an alternative amino acid (five additional variants) to achieve maximal repulsion between the DD and FADD. In detail, D228S causes disruption of a salt bridge with FADD R142, while Y291D leads to loss of hydrophobic interaction with L172, L176, V173, and N107. Figure 5 shows the structural localization of the selected mutations in the Fas/FADD complex structure.

**Figure 6:**
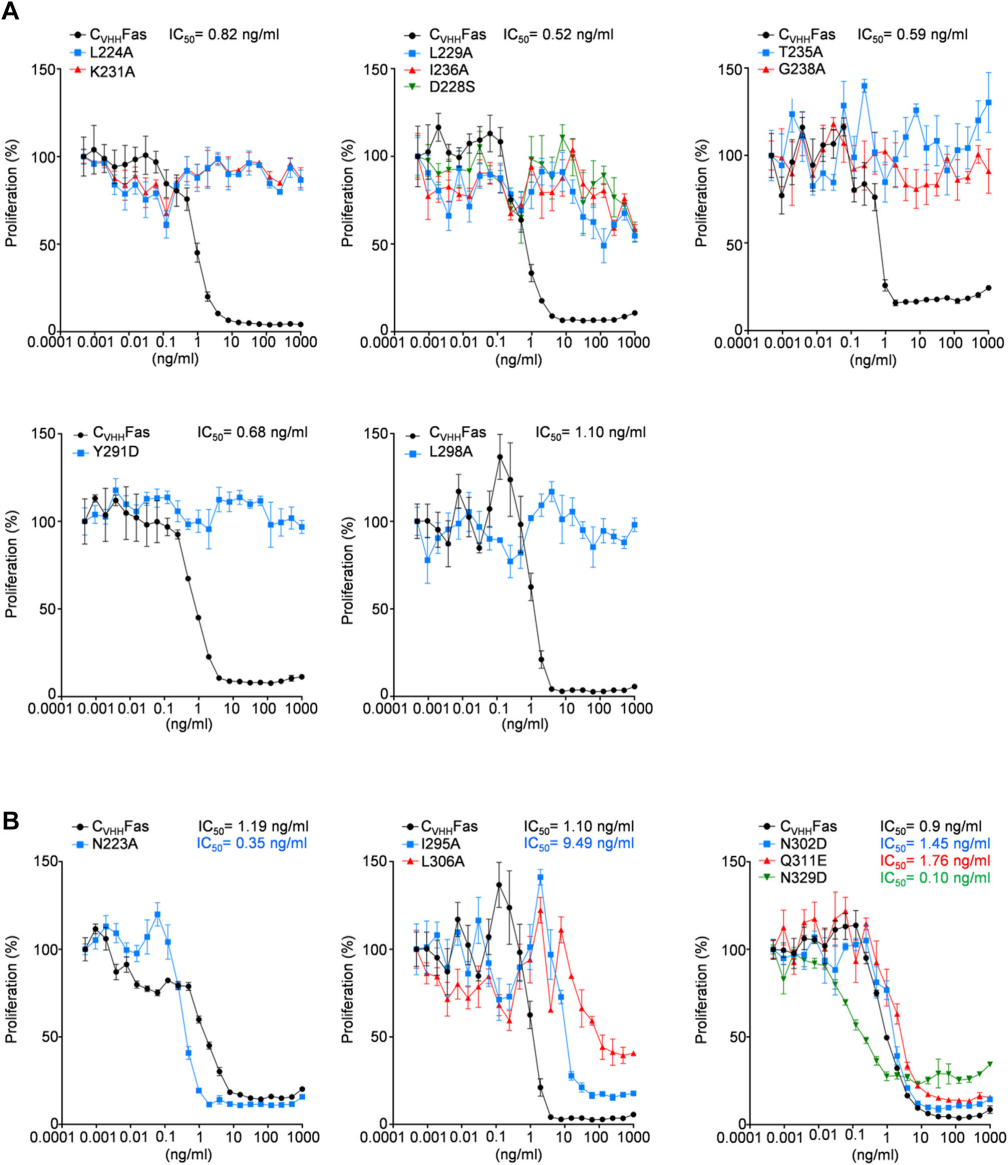
**Analysis of Fas structure-based mutations capacity to inhibit HIL-6–induced cellular proliferation.***A*, Ba/F3-gp130 cells expressing uncharacterized, LOF structure-based mutations (L224A, D228S, L229A, K231A, T235A, I236A, G238A, Y291D, and L298A) in C_VHH_Fas were treated with 20 ng/ml HIL-6 and increasing concentration of 3C (from 1 × 10^−4^ to 1 × 10^3^ ng/ml) for 72 h. *Black color*: Ba/F3-gp130-C_VHH_Fas cells; *blue* or *red* or *green color*: Ba/F3-gp130-C_VHH_Fas with indicated mutations. *B*, Ba/F3-gp130 cells expressing uncharacterized, GOF structure-based mutations (N223A, I295A, N302D, L306A, Q311E, N329D). Cells were treated as described above. *Black* color: Ba/F3-gp130-C_VHH_Fas cells; *blue* or *red* or *green* colors: Ba/F3-gp130-C_VHH_Fas with indicated mutations. Error bars indicate ± SEM. GOF, gain-of-function; HIL-6, Hyper-IL-6; LOF, loss-of-function.

The 15 structure-based mutations were introduced into the cDNA coding for C_VHH_Fas. Ba/F3-gp130 cells expressing the C_VHH_Fas variants were generated, and cell surface expression was verified by flow cytometry (Supporting information 1). Ba/F3-gp130-C_VHH_Fas (variant) cells were stimulated with 3C and HIL-6. The C_VHH_Fas alanine variants L224A, L229A, K231A, T235A, I236A, G238A, L298A and the two additional variants D228S, Y291D failed to inhibit HIL-6–induced proliferation, suggesting that these mutations cause LOF (Fig. 6*A*). On the other hand, N223A, I295A, L306A and N302D, Q311E, N329D still prevented cellular proliferation. Of these, I295A and L306A showed reduced inhibitory activity considering that higher concentrations of 3C were needed to abrogate cellular proliferation (IC_50_ = 9.49 ng/ml and not to be determined). Moreover, N223A and N329D were more effective (IC_50_ = 0.35 ng/ml and IC_50_= 0.10 ng/ml) and might represent mild GOF mutations (Fig. 6*B*).

In summary, 9 out of 15 SNPs are complete LOFs (L224A, L229A, K231A, T235A, I236A, G238A, L298A, D228S, Y291D), two are mild LOFs (I295A and L306A), two indicated no effect (N302D, Q311E), and two are proved to be GOFs (N223A and N329D).

### LOF SNPs and mutations are unable to induce apoptosis

**Figure 7:**
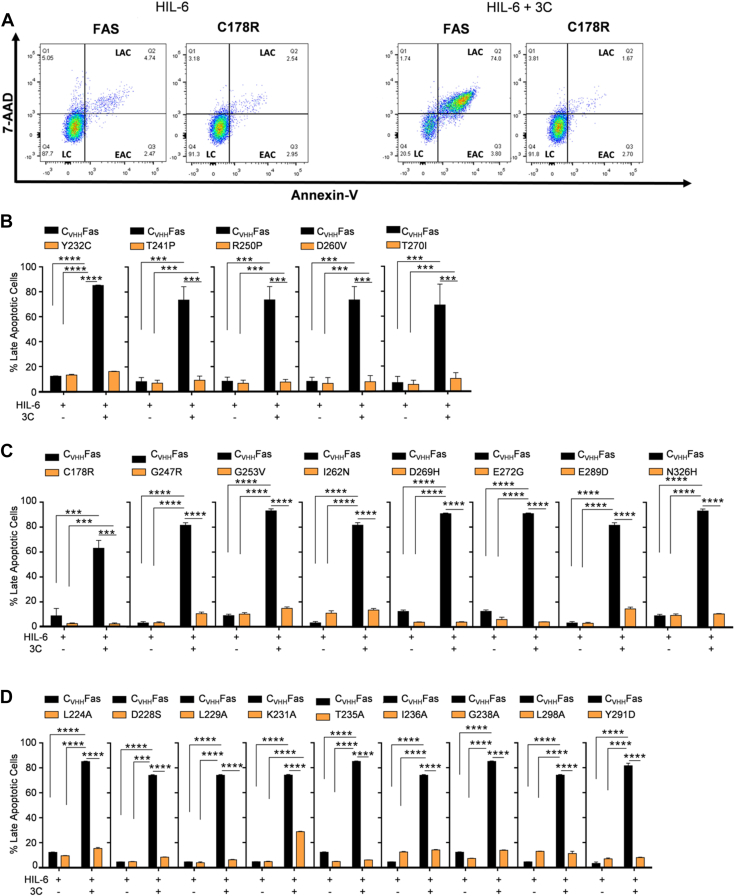
**LOF variants failed to induce apoptosis upon synthetic ligand stimulation.***A*, representative plots of apoptosis progression in Ba/F3-gp130-C_VHH_Fas and Ba/F3-gp130-C_VHH_Fas-C178R, LOF SNP, stimulated for 24 h with HIL-6 with and without 3C. Cells were stained with 7-AAD and Annexin-V and analyzed by flow cytometry. Graphics of apoptosis progression of Ba/F3-gp130-C_VHH_Fas WT compared to (*B*) previously published SNPs (Y232C, T241P, R250P, D260V, T270I), (*C*) uncharacterized, LOF SNPs (G247R, G253V, I262N, D269H, E272G, E289D, and N326H), and (*D*) uncharacterized, LOF structure-based mutations (Y232C, L224A, D228S, L229A, K231A, T235A, I236A, G238A, Y291D, and L298A). Error bars indicate ± SEM. ∗∗∗*p* < 0.001; ∗∗∗∗*p* < 0.0001. EAC, early apoptotic cells; HIL-6, Hyper-IL-6; LAC, late apoptotic cells; LC, living cells.

To analyze apoptosis progression in detail, we performed flow cytometry staining of 7-AAD and Annexin-V of Ba/F3-gp130 cells expressing C_VHH_Fas variants after 24 h stimulation with HIL-6 alone or in combination with the synthetic Fas ligand 3C. As illustrated in the schematic plots (Fig. 7*A*), the addition of HIL-6 resulted in 87.7% living Ba/F3-gp130-C_VHH_Fas cells, whereas co-incubation with 3C resulted in 74% apoptotic cells. The previously characterized SNP variants Y232C, T241P, R250P, D260V, T270I failed to induce apoptosis (Fig. 7*B* and Supporting information 2). A similar picture was seen for the eight ClinVar and Provean SNPs C178R, G247R, G253V, I262N, D269H, E272G, E289D, N326H (Fig. 7*C* and Supporting informations 3 and 4) and for the nine structure-based mutants L224A, D228S, L229A, K231A, T235A, I236A, G238A, L298A, Y291D, which all failed to inhibit proliferation with synthetic ligand stimulation and also did not induce apoptosis (Fig. 7*D* and Supporting informations 5 and 6). K231A was the only partial LOF alanine mutant with still 29% late apoptotic cells after 3C treatment *versus* 5% cells in the late apoptotic state without 3C, albeit this variant was not able to inhibit the proliferation of Ba/F3-gp130 cells.

**Figure 8:**
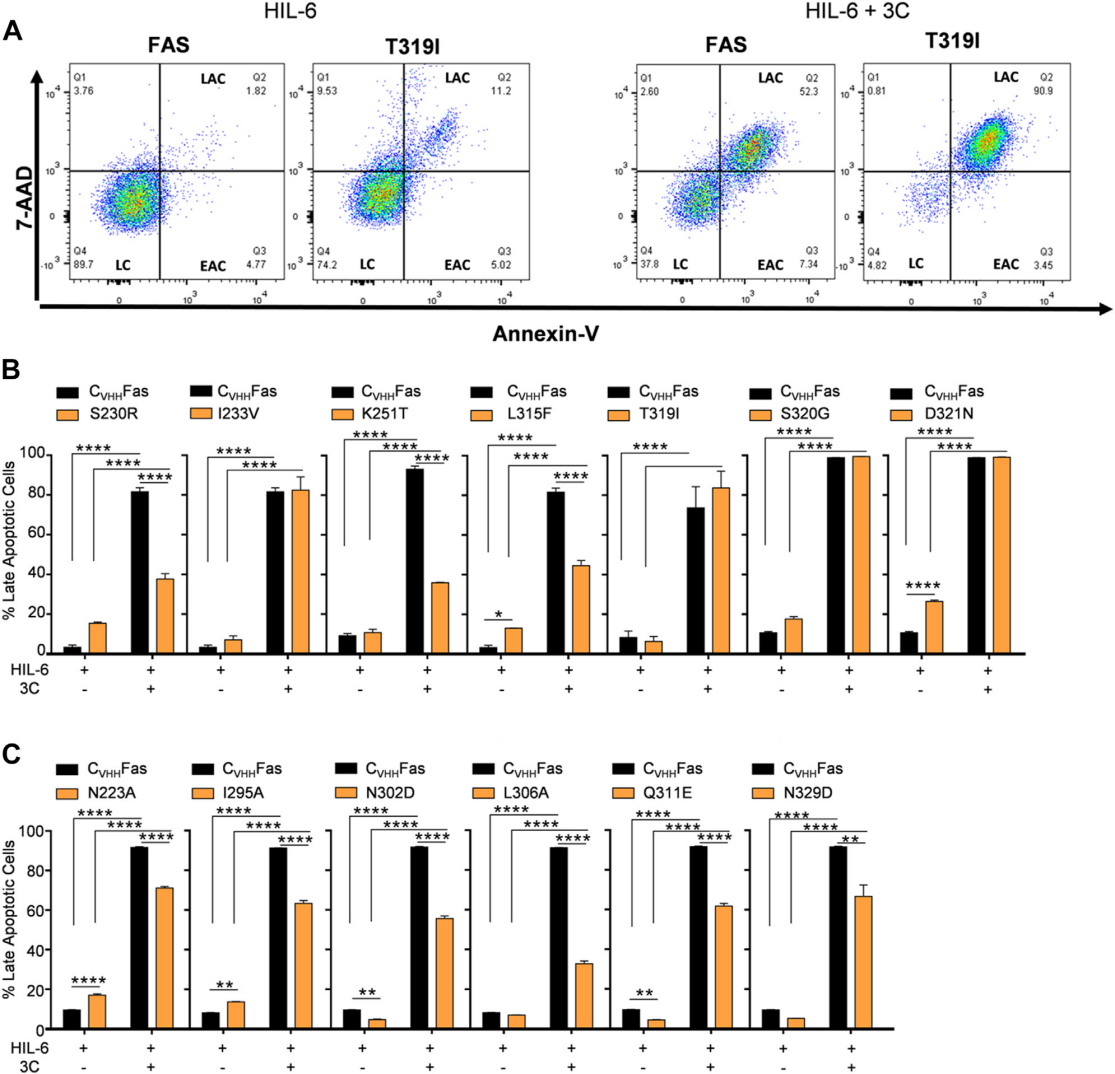
**Active variants were still able to induce apoptosis upon synthetic ligand stimulation.***A*, representative plots of apoptosis progression in Ba/F3-gp130-C_VHH_Fas and Ba/F3-gp130-C_VHH_Fas-T319I, GOF SNP, stimulated for 24 h with HIL-6 with and without 3C. Cells were stained with 7-AAD and Annexin-V and analyzed by flow cytometry. Graphics of apoptosis progression of Ba/F3-gp130-C_VHH_Fas WT compared to (*B*) uncharacterized, active SNPs (S230R, I233V, K251T, L315F, T319I, S320G, and D321N) and (*C*) uncharacterized, active structure-based mutations (N223A, I295A, N302D, L306A, Q311E, N329D). Error bars indicate ± SEM. ∗∗*p* < 0.01; ∗∗∗∗*p* < 0.0001. GOF, gain-of-function; HIL-6, Hyper-IL-6; C, living cells; LAC, late apoptotic cells; LEAC, early apoptotic cells.

Four mutations that indicated GOF (I233V, D321N) or with no effect (T319I, S320G) in the proliferation assays efficiently produced late apoptotic cells (82%, 83%, 99%, 99%, respectively). Of note, the remaining three variants (S230R, K251T (no effect), L315F) displayed fewer late apoptotic cells (37%, 36%, 44%, respectively), from which S230R and L315F were also seen in the proliferation assay to be mild LOF variants (Fig. 8, *A* and *B* and Supporting information 7).

The proliferation-inhibiting structure-based mutants N223A, I295A, N302D, L306A, Q311E, and N329D all induced cellular apoptosis (Fig. 8*C* and Supporting information 8). In detail, N223A, I295A, L306A, N302D, Q311E, and N329D induced 71%, 63%, 56%, 33%, 62%, and 67% late apoptotic cells, respectively.

The apoptotic assays generally confirmed LOF phenotypes for the previously described SNPs Y232C, T241P, R250P, D260V, T270I and the ClinVar and Provean predicted SNPs C178R, G247R, G253V, I262N, D269H, E272G, E289D, N326H and the structure-based mutations L224A, D228S, L229A, K231A, T235A, I236A, G238A, L298A, Y291D.

Taken together, a consistent milder LOF phenotype was seen in proliferation and apoptosis assays for the SNPs S230R and L315F and the structure-based mutations I295A, L306A and a comparable to WT Fas phenotype for the SNPs T319I and S320G.

### Capacity of Fas-SyCyR variants to activate caspases 3 and 7

**Figure 9:**
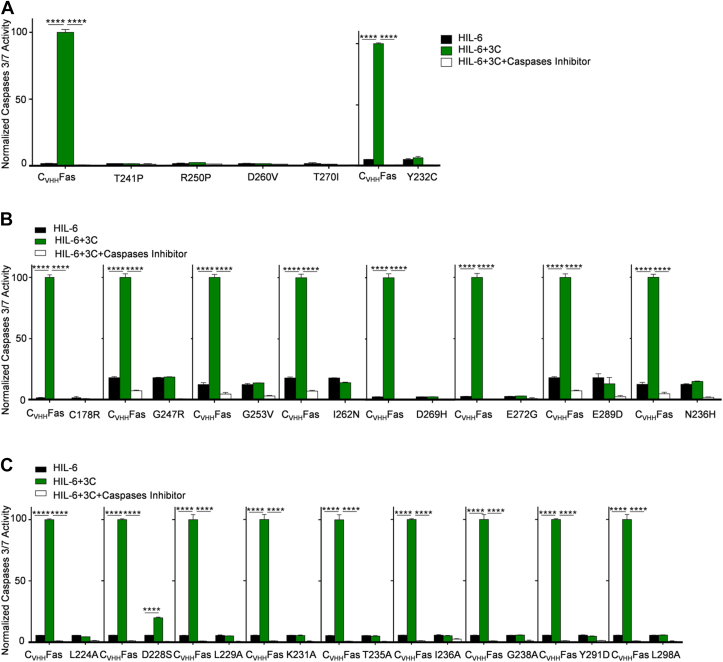
**Caspases 3/7 activation was not detected in Ba/F3-gp130 expressing C**_**VHH**_**Fas with LOF mutations.** Activation of caspases 3/7 in Ba/F3-gp130-C_VHH_Fas WT and with (*A*) LOF, previously published SNPs (Y232C, T241P, R250P, D260V, T270I), (*B*) uncharacterized, LOF SNPs (C178R, G247R, G253V, I262N, D269H, E272G, E289D, and N326H), and (*C*) uncharacterized, LOF structure-based mutations (L224A, D228S, L229A, K231A, T235A, I236A, G238A, Y291D, and L298A). Cells were stimulated for 6 h with only HIL-6 (*black bars*), plus 3C (*green bars*), plus caspases inhibitor (*white bars*). Error bars indicate ± SEM. ∗∗∗∗*p* < 0.0001. HIL-6, Hyper-IL-6.

An early hallmark of apoptosis is the activation of the effector caspases 3 and 7 (31), which were analyzed by a quantitative, fluorescent caspase-cleavage assay. Ba/F3-gp130 cells expressing C_VHH_Fas variants were stimulated for 6 h with HIL-6 in the presence and absence of the synthetic Fas ligand 3C. Caspase 3/7 activation after the addition of 3C in Ba/F3-gp130 cells expressing C_VHH_Fas was set to 100%, and the efficiency of the other C_VHH_Fas variants was calculated accordingly. As observed for the inhibition of proliferation and determination of late apoptotic cells, the five previously characterized LOF SNPs, Y232C, T241P, R250P, D260V, and T270I completely failed to induce caspase 3/7 activation (Fig. 9*A*). Likewise, the eight other identified LOF SNPs from ClinVar and Provean C178R, G247R, G253V, I262N, D269H, E272G, E289D, and N326H (Fig. 9*B*) failed to induce caspase 3/7. Eight out of the nine others identified structure-based LOF mutations L224A, L229A, K231A, T235A, I236A, G238A, Y291D, and L298A (Fig. 9*C*) were unable to activate the effector caspases, although D228S still resulted in about 20% activation (Fig. 9*C*). D228S causes the loss of a salt bridge interaction between Fas and FADD. In the D228S, the serine is still within 4.5 Å distance to R142 of FADD and may hence be able to form a H-bond which does not completely abrogate the interaction but significantly weaken it.

**Figure 10:**
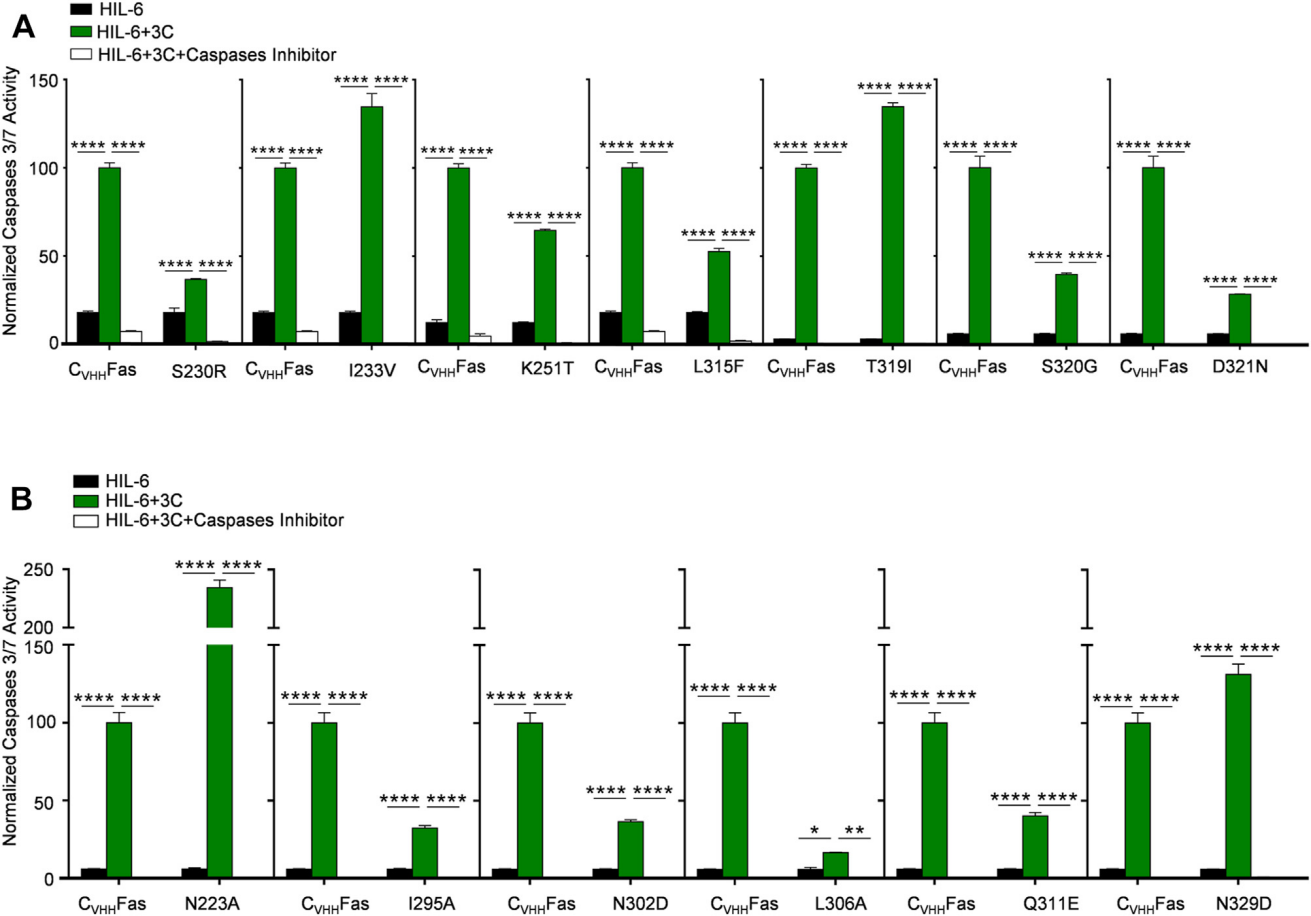
**Detection of caspases 3/7 activation in Ba/F3-gp130 expressing C**_**VHH**_**Fas with GOF mutations.** Activation of caspases 3/7 in Ba/F3-gp130-C_VHH_Fas WT and with (*A*) uncharacterized, active SNPs (S230R, I233V, K251T, L315F, T319I, S320G, and D321N), (*B*) uncharacterized, active structure-based mutations (N223A, I295A, N302D, L306A, Q311E, N329D). Cells were stimulated for 6 h with only HIL-6 (*black bars*), plus 3C (*green bars*), plus caspases inhibitor (*white bars*). Error bars indicate ± SEM. ∗*p* < 0.5; ∗∗*p* < 0.01; ∗∗∗∗*p* < 0.0001. GOF, gain-of-function; HIL-6, Hyper-IL-6.

Compared to C_VHH_Fas, induction of caspase 3/7 of the active SNPs S230R, I233V, K251T, L315F, T319I, S320G, D321N was 37%, 134%, 64%, 52.5%, 135%, 39% and 28%, respectively (Fig. 10*A*).

Similarly, the structure-based mutations N223A, I295A, N302D, L306A, Q311E, and N329D induced caspase 3/7 as well, albeit with different efficiencies compared to stimulation of cells expressing the WT receptor (Fig. 10*B*). In detail, the effector caspase activation of N223A and N329D mutants supported the previous proliferation and late apoptosis data as them as novel GOF mutants, with a respective percentage equal to 234% and 131% compared to C_VHH_Fas, respectively. On the contrary, I295A, N302D, L306A, and Q311E induced 32%, 36%, 17%, and 40%, respectively, resulting in mild LOF mutations.

We have summarized all findings in Tables 1 and 2. The five previously described LOF SNPs Y232C, T241P, R250P, D260V, and T270I were also in our experiments unable to inhibit proliferation, to induce cellular apoptosis and to activate effector caspases 3 and 7. The same picture was seen for the ClinVar-reported and Provean-predicted SNPs of the transmembrane and intracellular Fas domain C178R, G247R, G253V, I262N, D269H, E272G, E289D, and N326H and the structure-based L224A, L229A, K231A, T235A, I236A, G238A, Y291D, L298A mutants, with 19% activation for D228S. Contrary, the SNPs S230R, I233V, K251T, L315F, T319I, S320G, D321N and the designed mutations L224A, D228S, L229A, K231A, T235A, I236A, G238A, Y291D, L298A were still able to abrogate proliferation, to induce cellular apoptosis, and to activate effector caspases to some degree.

**Table 1:** Summary of selected Fas SNPs and activity

	ClinVar rating	ClinVar disease	Provean prediction	Inhibition of proliferation	(%) Late apoptosis	Caspases 3/7	Summary activity (number of criteria fulfilled out of 3)
Previously published SNPs							
Y232C	Pathogenic, uncertain significance	ALPS	Not tested	None	None	None	LOF (3/3)
T241P	Pathogenic	ALPS	Not tested	None	None	None	LOF (3/3)
R250P	Likely pathogenic	ALPS	Not tested	None	None	None	LOF (3/3)
D260V	Pathogenic	ALPS	Not tested	None	None	None	LOF (3/3)
T270I	Pathogenic	ALPS	Not tested	None	None	None	LOF (3/3)
Previously uncharacterized SNPs							
C178R	Pathogenic	SCC	Not tested	None	None	None	LOF (3/3)
S230R	-	-	Yes	IC_50_ = 31 ng/ml	37%	37%	Reduced (3/3)
I233V	-	-	Yes	IC_50_ = 0.11 ng/ml	82%	134%	GOF (2/3)
G247R	-	-	Yes	None	None	None	LOF (3/3)
K251T	-	-	Yes	IC_50_ = 0.91 ng/ml	36%	64%	Reduced (2/3)
G253V	Uncertain significance	ALPS	Not tested	None	None	None	LOF (3/3)
I262N	Likely-pathogenic, uncertain significance	-	Not tested	None	None	None	LOF (3/3)
D269H	-	-	Yes	None	None	None	LOF (3/3)
E272G	Uncertain significance	-	Not tested	None	None	None	LOF (3/3)
E289D	Uncertain significance	ALPS	Not tested	None	None	None	LOF (3/3)
L315F	Uncertain significance	ALPS	Not tested	IC_50_ = 29.8 ng/ml	44%	52.5%	Reduced (3/3)
T319I	Uncertain significance	ALPS	Not tested	IC_50_ = 0.95 ng/ml	83%	135%	GOF (2/3)
S320G	Likely benign	ALPS	Not tested	IC_50_ = 2.64 ng/ml	99%	39%	Reduced (1/3)
D321N	Uncertain significance	ALPS	Not tested	IC_50_ = 0.20 ng/ml	99%	28%	GOF (1/3)
N326H	Uncertain significance	-	-	None	None	None	LOF (3/3)

**Table 2:** Summary of Fas structure-based mutations activity

Structure-based mutations	Inhibition of proliferation	(%) Late apoptosis	(%) Caspases 3/7	Summary activity (number of criteria fulfilled out of 3)
N223A	IC_50_ = 0.35 ng/ml	71%	234%	GOF (2/3)
L224A	None	None	None	LOF (3/3)
D228S	None	None	19%	LOF (2/3)
L229A	None	None	None	LOF (3/3)
K231A	None	29%	None	LOF (2/3)
T235A	None	None	None	LOF (3/3)
I236A	None	None	None	LOF (3/3)
G238A	None	None	None	LOF (3/3)
Y291D	None	None	None	LOF (3/3)
I295A	IC_50_ = 9.49 ng/ml	63%	32%	Reduced (3/3)
L298A	None	None	None	LOF (3/3)
N302D	IC_50_ = 1.45 ng/ml	56%	36%	Reduced (2/3)
L306A	Not detectable IC_50_	33%	17%	Reduced (3/3)
Q311E	IC_50_ = 1.76 ng/ml	62%	40%	Reduced (2/3)
N329D	IC_50_ = 0.10 ng/ml	67%	131%	GOF (2/3)

### Combination of mutations to strengthen GOF and LOF

One plausible application for SyCyRs could be to strengthen chimeric antigen receptor (CAR) T-cell therapies, either supporting or suppressing the activity of engineered T-cells. Following this aim, we were curious to test if combining GOF or LOF mutations would result in stronger and weaker activation of synthetic WT Fas.

**Figure 11:**
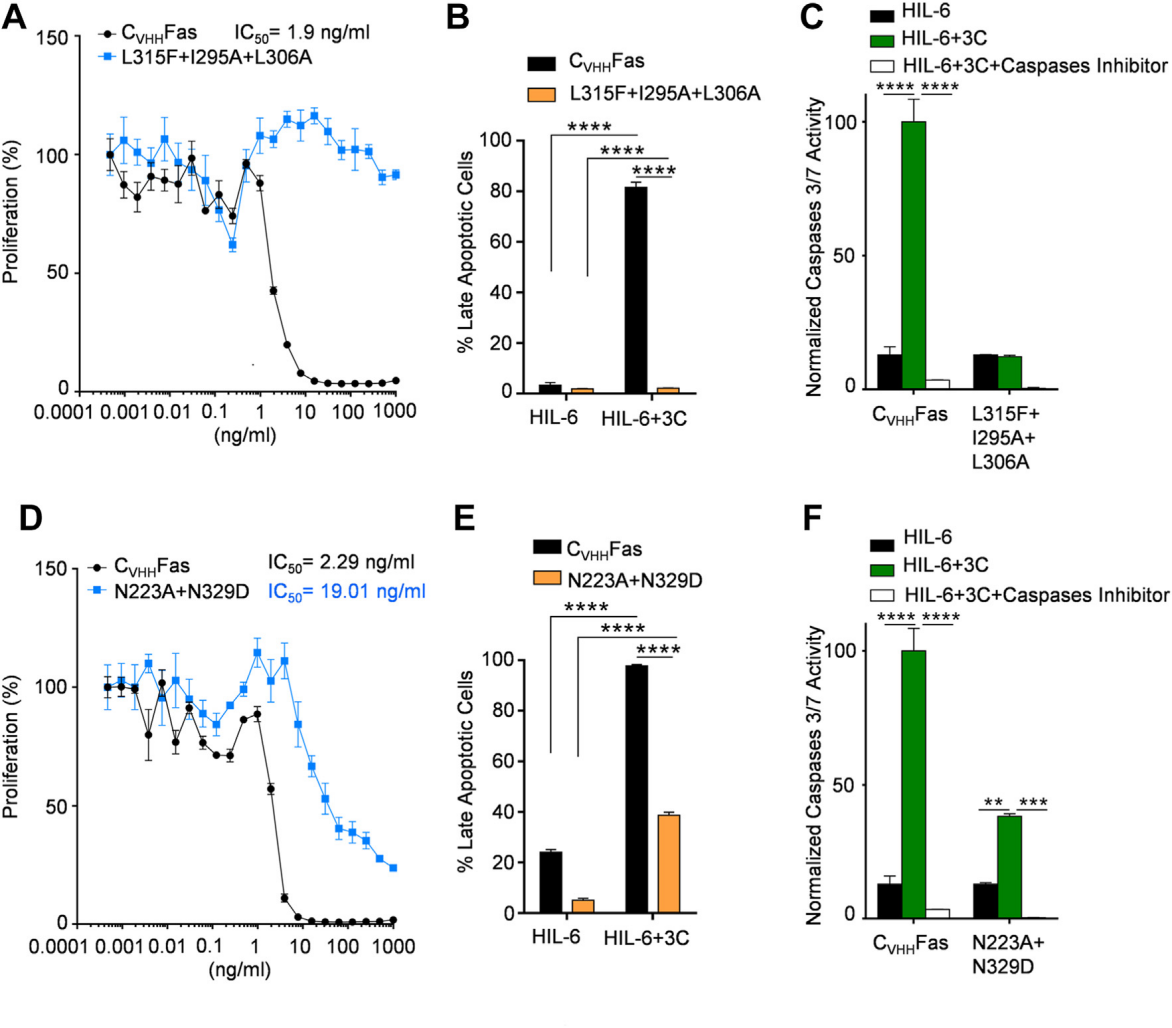
**Combination of selected mutations to achieve more severe phenotypes.** Proliferation assay of (*A*) Ba/F3-gp130-C_VHH_Fas-L315F+I295A+L306A and (*D*) Ba/F3-gp130-C_VHH_Fas-N223A+N329D. Cells were treated with 20 ng/ml HIL-6 and increasing concentration of 3C (from 1 × 10^−4^ to 1 × 10^3^ ng/ml) for 72 h. Apoptosis progression in (*B*) Ba/F3-gp130-C_VHH_Fas-L315F+I295A+L306A and (*E*) Ba/F3-gp130-C_VHH_Fas-N223A+N329D. Cells were stimulated for 24 h with HIL-6 with and without 3C. Cells were stained with 7-AAD and Annexin-V and analyzed by flow cytometry. Activation of caspases 3/7 in (*C*) Ba/F3-gp130-C_VHH_Fas-L315F+I295A+L306A and (*F*) Ba/F3-gp130-C_VHH_Fas-N223A+N329D. Cells were stimulated for 6 h with only HIL-6 (*black bars*), plus 3C (*green bars*), plus caspases inhibitor (*white bars*). Error bars indicate ± SEM. ∗∗*p* < 0.01; ∗∗∗*p* < 0.001; ∗∗∗∗*p* < 0.0001. HIL-6, Hyper-IL-6.

The SNP L315F and the alanine mutants I295A and L306A had consistently reduced Fas activity in our assays. Therefore, we combined them in a triple mutant to analyze if this will result in a more severe LOF phenotype. As expected, the combination of the mild LOF L315F, I295A, and L306A in one variant resulted in complete LOF, since this variant was unable to inhibit the proliferation of Ba/F3-gp130 cells (Fig. 11*A*), did not induce late apoptosis (Fig. 11*B* and Supporting information 9), and failed to activate caspases 3 and 7 (Fig. 11*C*).

On the other hand, the mutants N223A and N329D alone conferred a stronger capacity to inhibit proliferation, partially reduced amounts of late apoptotic cells, and significantly higher caspase 3/7 activity compared to WT, after activation with synthetic Fas. Hence, likewise, we combined those two mutants to analyze if this will result in a stronger GOF phenotype. Unexpectedly, the combination of two stronger activated mutations N223A and N329D resulted in a partial LOF variant with a lower capacity to inhibit proliferation (IC_50_ = 19.01 ng/ml) (Fig. 11*D*), to induce apoptosis (38% of late apoptotic cells *versus* 98% for WT Fas) (Fig. 11*E* and Supporting information 9), and to induce effector caspases activation with a percentage equal to 38% (Fig. 11*F*).

In conclusion, the combination of three less active mutations (L315F, I295A, L306A) resulted in a complete LOF phenotype, while the combination of two GOF mutations (N223A plus N329D) did not lead to a more active variant.

### Dominant-negative effect of selected Fas single nucleotide mutations

**Figure 12:**
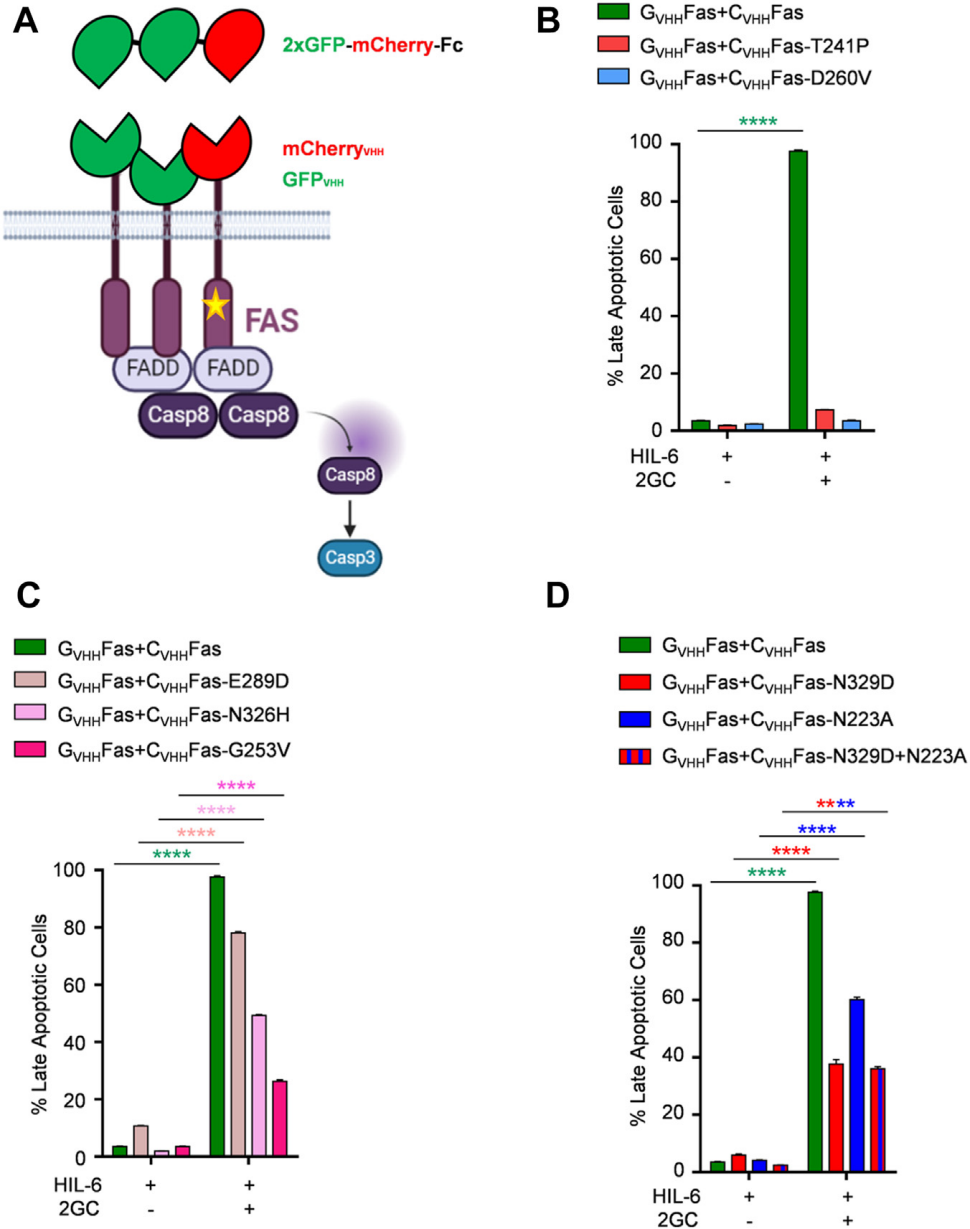
**Verification of the dominant-negative effect of Fas selected mutation.***A*, schematic representation of synthetic C_VHH_Fas-mutated and G_VHH_Fas complex formation and activation by synthetic ligand 2GC. Apoptosis progression in (*B*) Ba/F3-gp130-G_VHH_Fas-C_VHH_Fas wt, Ba/F3-gp130-G_VHH_Fas-C_VHH_Fas-T241P, and Ba/F3-gp130-G_VHH_Fas-C_VHH_Fas-D260V; (*C*) Ba/F3-gp130-G_VHH_Fas-C_VHH_Fas wt, Ba/F3-gp130-G_VHH_Fas-C_VHH_Fas-E289D, Ba/F3-gp130-G_VHH_Fas-C_VHH_Fas-N326H, and Ba/F3-gp130-G_VHH_Fas-C_VHH_Fas-G253V; and in (*D*) Ba/F3-gp130-G_VHH_Fas-C_VHH_Fas wt, Ba/F3-gp130-G_VHH_Fas-C_VHH_Fas-N329D, Ba/F3-gp130-G_VHH_Fas-C_VHH_Fas-N223A, and Ba/F3-gp130-G_VHH_Fas-C_VHH_Fas-N329D+N223A. Cells were stimulated for 24 h with HIL-6 with and without 2GC. Cells were stained with 7-AAD and Annexin-V and analyzed by flow cytometry. Error bars indicate ± SEM. ∗∗∗∗*p* < 0.0001. HIL-6, Hyper-IL-6.

ALPS patients have been mainly associated with missense mutations in FAS gene, which cause the disruption of the apoptotic pathway by dominant-negative interference (29). Normally, following endogenous ligand binding, Fas is pre-assembled as homotrimeric receptor. At least one copy of mutated Fas protein (heterozygous) can cause a dysfunctional trimeric receptor complex (32). In order to test the dominant-negative effect of selected Fas mutations from this study, we retrovirally transduced Ba/F3-gp130-C_VHH_Fas WT and mutant cells with an additional synthetic G_VHH_Fas cDNA (Fig. 12*A*). Cell surface expression of G_VHH_Fas was verified by flow cytometry using Myc-tag antibodies (Supporting information 10). Next, cells were stimulated for 24 h by synthetic fusion proteins composed of two GFPs and one mCherry (2GC), in order to induce receptor trimerization of two WT and one mutated synthetic Fas receptor (Fig. 12*A*). We tested the two previously described LOF mutations T241P and D260. As shown in Figure 12*B* (Supporting information 11), Ba/F3-gp130 cells expressing C_VHH_Fas T241P or D260 plus G_VHH_Fas were unable to induce apoptosis by heterotrimerization, confirming the dominant-negative effect of these two variants. We proceeded with testing three previously uncharacterized nonsynonymous SNPs (E289D, N326H, and G253V), which were LOF in our characterization. Interestingly, those mutations still impaired apoptosis compared to normal Fas but with a percentage of apoptotic cells equal to 78%, 49%, and 26%, respectively (Fig. 12*C* and Supporting information 12), thus having a weaker dominant-negative effect. Finally, we tested the effect of the GOF structure-based mutations that initially resulted in weaker GOF (N329D, N223A, and N329D+N223A). After stimulation, we obtained, respectively, 37.6%, 60%, and 36% of apoptosis (Figs. 12*D* and S13), also showing reduced Fas function as heterotrimers.

## Discussion

Synthetic biology has become an engrossing alternative option to analyze cytokine signal transduction as well as for the development of personalized therapies (5), including the approval of CAR T-cell immunotherapy for acute lymphoblastic leukemia as the first gene therapy (33). Initially, we generated fully synthetic cytokine receptors to phenocopy prototypical dimeric cytokine receptors for IL-6, IL-22, and IL-23 (10, 11) and recently adopted this system for the activation of trimeric receptors for TNF and FasL (6). The SyCyRs for the death receptor Fas efficiently activated cellular apoptosis as shown by inhibition of cellular proliferation, Annexin-V staining, and caspase 3 cleavage assays (6). The specificity of induced apoptosis due to the synthetic Fas activation was verified by a previously described LOF mutation in the DD (6), which was associated with the development of ALPS (34).

In this study, we used Fas-SyCyR as a systematic tool to characterize the functional and molecular mechanism of LOF or (partial) GOF mutations associated with Fas, with or without a direct immune diseases correlation. We initially predicted relevant SNPs found in the transmembrane and intracellular human Fas domain by in-depth structure-guided analysis (16) or by basic online data processing tools such as Provean (17). Subsequently, we selected 20 SNPs and 15 structure-based mutations. We showed that none of the 35 mutation results in global instability of the protein, since all were normally expressed on the cell surface, confirming previous data reporting expression abnormalities mainly for extracellular Fas mutations (29, 30).

To date, most described LOF SNPs in Fas were found in the transmembrane and intracellular DD and presented a more pronounced disrupted phenotype and defective apoptosis in lymphocytes in patients affected by ALPS and SCC, compared to extracellular Fas SNPs (26). Furthermore, patient LOF mutations in the DD of Fas are typically heterozygous because homozygosity would be lethal (25). To validate our system, we selected five SNPs previously associated to ALPS and molecularly described as LOF mutations. Bettinardi *et al*. showed how two ALPS-affected siblings were carrying the same mutation Y232C in Fas gene causing a defect in apoptosis (23), while patients carrying Fas SNPs T241P, R250P, and D260V showed reduced apoptosis (26, 28), reduced T-cell loss (25), and altered death-inducing–signaling complex formation (29). Lastly, the SNP T270I exhibited inhibition of Fas-mediated apoptosis deduced by higher cell viability and absence of FADD recruitment (30). Our results confirmed and completed the previous studies with additional structural analysis, apoptosis, and caspases 3 and 7 cleavage assays. We further shed light on the molecular mechanism of 15 additional new SNPs reported in ClinVar as (likely) pathogenic or with uncertain significance or predicted by Provean. From our analysis, eight SNPs resulted in complete LOF, four showed a partially reduced Fas activity, while three conferred a milder GOF. We widely demonstrated that prediction by in-depth structure-guided analysis or by basic online data processing tools such as Provean may be useful as preliminary screening but need to be experimentally and systematically validated. We predicted 15 further LOF candidates using structure-based modeling. To design LOF variants, DD residues within the DD-FADD interface were substituted by alanine (ten alanine variants) or an alternative amino acid (five rational designed variants). N223A and N329D did not negatively affect Fas-induced inhibition of proliferation, apoptosis, and caspase activation, while I295A, N302D, L306A, and Q311E only partially diminished the WT activity.

SyCyRs might potentially be used to boost CAR T-cell therapy (6). Despite the significant clinical success of CAR T-cells in treating patients with leukemia and lymphoma, two primary clinical side effects have been encountered: cytokine release syndrome and neurotoxicity (35). We propose a plausible innovative approach to overcome the side effects by promoting or repressing the activity of transgenic T-cells, respectively activating gp130 and synthetic Fas. In line with this aim, we were interested to test if combining GOF or LOF mutations would produce greater or weaker activation of synthetic WT Fas, in order to induce an improved (stronger or weaker) synthetic Fas activation in primary T-cells and consequent suppression of the deadly pro-inflammatory response.

We observed that two more intensely activated mutations, N223A and N329D, were combined to create a stronger active variant; however, the combination was unfavorable and resulted in a mild LOF phenotype. N223 is in close proximity to N136 and R135 of FADD, but in the structure, it seems to not be in contact with FADD. N329 is far away from FADD. Looking at the structure, we cannot explain the LOF phenotype of the N223A and N329D combination. Possibly, the exchange of both amino acids might lead to a decreased stability of the Fas, since these two amino acids contribute to the overall protein stability with −9.7 KJ/mol (N223) and −32.1 KJ/mol (N329) in the closed conformation (PDB: 1DDF) and −86.3 KJ/mol (N329) in the open conformation (PDB: 3EZQ) (36).

To conclude, we aimed at testing the dominant-negative effect of some selected SNPs and mutations that was confirmed for two ALPS-associated LOF SNPs and partially showed for three additional uncharacterized nonsynonymous SNPs and two GOF structure-based mutations, as well as the combination of the two.

Indubitably, following this initial screening, one possible prospective analysis is to test the selected disease-related SNPs and mutations in primary immune cells that co-express endogenous Fas activated by FasL.

In summary, defining the specific molecular mechanism that cause defective apoptotic pathway using a systematic and rapid tool such as Fas-SyCyR that supports *in silico* analysis can help to better understand Fas pathological roles and to establish suitable therapeutic strategies. Moreover, our Fas-SyCyR system may have a useful role in enhancing the application of synthetic biology for personalized therapies.

## Experimental procedures

### Cell culture

Murine Ba/F3-gp130 cells were obtained from Immunex. Ba/F3-gp130 and Ba/F3-gp130-C_VHH_Fas cells were cultured at 37 °C with 5% CO_2_ in a water-saturated atmosphere in Dulbecco’s modified Eagle’s Medium high glucose culture medium (GIBCO, Life Technologies) with 10% fetal calf serum (GIBCO, Life Technologies,), 60 mg/l penicillin, and 100 mg/l streptomycin (Genaxxon Bioscience GmbH), and supplemented with Hyper-IL-6, a fusion protein of IL-6 and soluble IL-6 receptor (37). In detail, 0.2% (10 ng/ml) of conditioned medium was used from a stable clone of CHO-K1 cells secreting Hyper-IL-6 in the supernatant (stock solution approximately 10 μg/ml as determined by ELISA).

### Transduction and selection of cells

According to a prior description (38), Fas-SyCyR–coding pMOWS plasmids were used to retrovirally transduce Ba/F3-gp130 cells. The packing cell line was Phoenix-Eco cells (received from Ursula Klingmüller (DKFZ)). After transduction, cells were expanded as above described plus hygromycin B (1 mg/ml) (Carl Roth).

### Cell surface detection of synthetic cytokine receptors

α-HA-tag mAb (C29F4; cat. #S724S; Cell Signaling Technology; dilution 1:1000) was used to identify the expression of Fas-SyCyR (WT and all variants) in the transfected Ba/F3-gp130 cells. The cells were resuspended in 50 μl of fluorescence-activated cell sorting (FACS) solution plus primary antibody HA after being washed in FACS buffer (PBS, 1% bovine serum albumin). Cells were washed and resuspended in 50 μl FACS buffer with secondary antibody Alexa Fluor 488–conjugated Fab goat anti-rabbit IgG (cat. # A11070; 1:500) before being incubated for at least 1 h at room temperature. Cells were washed, resuspended in 200 μl of FACS buffer, and the fluorescence signal was acquired by flow cytometry (BD FACSCanto II flow-cytometer, BD Biosciences). Data were analyzed with FlowJo V10 (https://www.flowjo.com/solutions/flowjo/downloads) (FlowJo LLC).

### Proliferation assay

Ba/F3-gp130 cells were washed three times with PBS. 5 × 10^4^ cells were suspended in Dulbecco’s modified Eagle’s Medium containing 10% FCS, 60 mg/l penicillin, and 100 mg/ml streptomycin. Cells were stimulated for 72 h in a volume of 100 μl with Hyper-IL-6 and increasing concentration of synthetic ligand 3xmCherry-Fc (from 1 × 10^−4^ to 1 × 10^3^ ng/ml). As previously described (6), the Cell Titer Blue Viability Assay (Promega) was used to determine the approximate number of viable cells by measuring the fluorescence (excitation 560 nm, emission 590 nm) using the Infinite M200 Pro plate reader (Tecan). After adding 20 μl per well of Cell Titer Blue reagent (point 0), fluorescence was measured approximately every 20 min for up to 2 h. For each condition of an experiment, four wells were measured. All values were normalized by subtracting time point 0 values from the final measurement. Average of values of Ba/F3-gp130-C_VHH_Fas WT cells stimulated with lowest concentration of 3xmCherry-Fc (1 × 10^−4^ ng/ml) was set as 100%, and the efficiency of the other C_VHH_Fas variants was calculated accordingly.

### Apoptosis assay

Ba/F3-gp130 cell lines were washed three times with PBS. 2.5 × 10^5^ cells were seeded and stimulated for 24 h with HIL-6 (20 ng/ml) with and without 3xmCherry-Fc (100 ng/ml). Afterward, cells were washed twice with ice-cold PBS and resuspended in 50 μl Annexin-V–binding buffer (BD Bioscience) and Annexin-V (1:600) (ImmunoTools). Following incubation at room temperature for 15 min in the dark, 1 μl 7-AAD (R&D Systems) was added and diluted in 200 μl of Annexin-V–binding buffer. Analysis was carried out by flow cytometry (BD FACSCanto II flow-cytometer, BD Biosciences). Data were evaluated using FlowJo V10 (FlowJo LLC).

### Caspases 3/7 activation assay

Effector caspases 3 and 7 activation was detected using Amplite Fluorimetric Caspase 3/7 Assay Kit ∗Green Fluorescence∗ (AAT Bioquest, cat. #13503). Briefly, Ba/F3-gp130 cell lines were washed three times with PBS. 1 × 10^5^ cells were seeded in 100 μl/well in 96 wells/plate and stimulated with 20 ng/ml HIL-6 with and without 100 ng/ml 3xmCherry-Fc. After 6 h stimulation, 1 μl of 1 mM caspase 3/7 inhibitor Ac-DEVD-CHO stock solution was added only in the selected samples for 10 min at room temperature, and subsequently, 100 μl of caspase 3/7 working solution was added for at least 1 h at room temperature. Fluorescence intensity was measured at Ex/Em= 490/525 nm. For each condition of an experiment, three wells were measured. All values were normalized by subtracting background values from the final measurement. Caspase 3/7 activation after addition of 3xmCherry-Fc in Ba/F3-gp130 cells expressing C_VHH_Fas was set to 100%, and the efficiency of the other C_VHH_Fas variants was calculated accordingly.

### Statistical analysis

Data are shown as mean ± SEM. Multiple comparisons were determined with GraphPad Prism 6 (GraphPad Software) using one-way ANOVA column analyses. Statistical significance was set to *p* < 0.05 (∗∗∗∗ *p* < 0.0001, ∗∗∗ *p*< 0.001, ∗∗ *p* < 0.01, ∗*p* < 0.05).

### Structural analysis

Structural analysis of FAS and FADD was performed using UCSF Chimera (39) based on PDB 3EZQ.

## Data availability

### Lead contact

Further information and requests for resources and reagents should be directed to and will be fulfilled by the lead contact, Jürgen Scheller (jscheller@uni-duesseldorf.de).

### Materials availability

This study did not generate new unique reagents. All cDNAs are available upon request.

### Limitation of study

As shown and discussed before, this study shows early research upon the usage of a new synthetic cytokine system. Further studies are necessary to verify these findings *in vivo* and to analyze the potential combination with the CAR T-cell treatment.

## Supporting information

This article contains supporting information.

## Conflict of interest

The authors declare that they have no conflicts of interest with the contents of this article.
