# Supplementary material for: Synthetic receptor platform to identify loss-of-function single nucleotide variants and designed mutants in the death receptor Fas/CD95
Source: J Biol Chem. 2023 Jun 29;299(8):104989. doi: 10.1016/j.jbc.2023.104989 (PMC10413154; doi:10.1016/j.jbc.2023.104989)
Supplement: Supporting information [file mmc1.pdf]

Supporting Information S-1

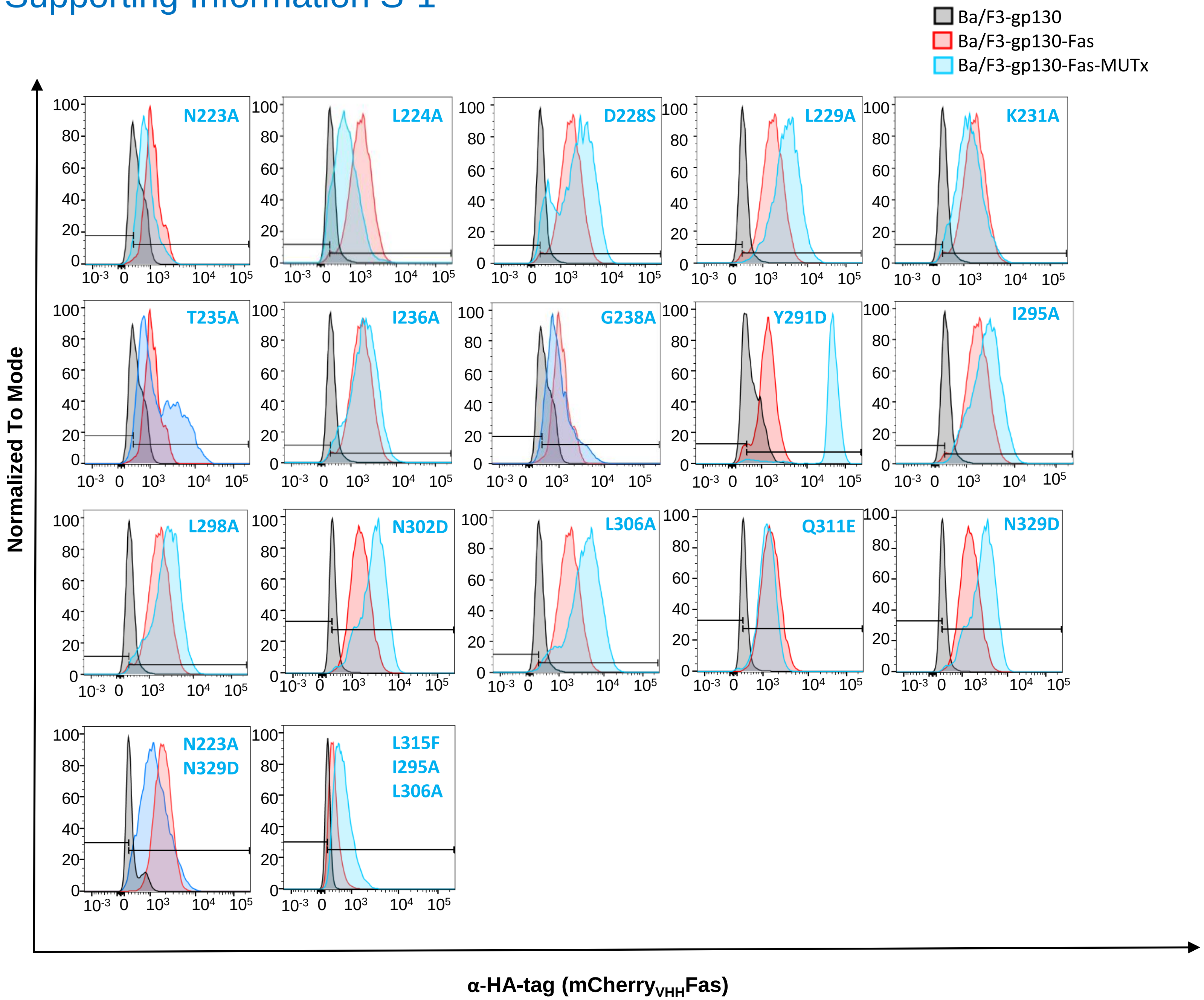

S-1: Cell surface expression of Ba/F3-gp130 (black), Ba/F3-gp130-C_{VHH}Fas and Ba/F3-gp130-C_{VHH}Fas with indicated mutations by specific detection of surface HA-tag through flow-cytometry.

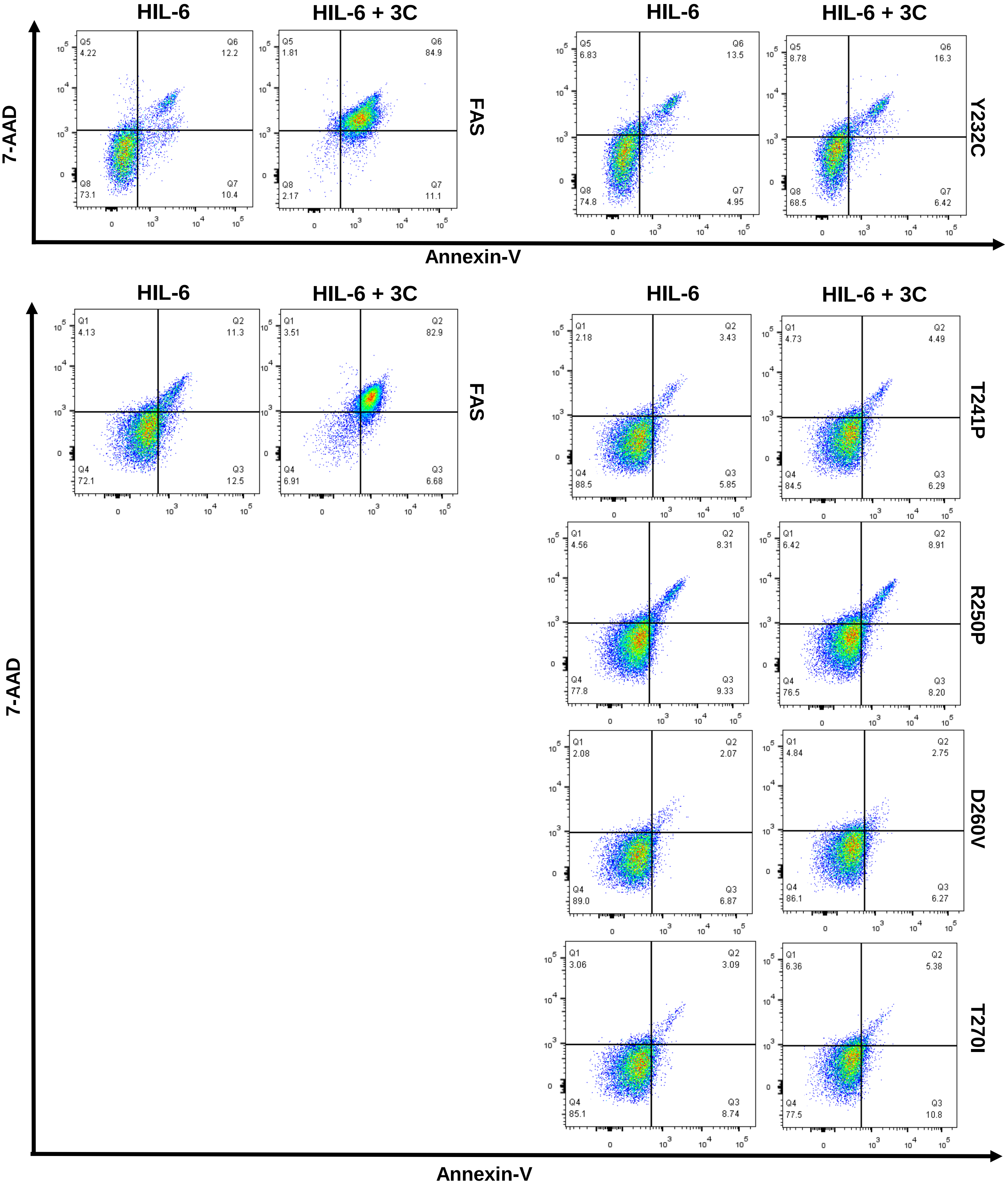

S-2: Flow-cytometry plots of apoptosis progression in Ba/F3-gp130 expressing C_{VHH}Fas wild-type and previously published SNPs (Y232C, T241P, R250P, D260V, T270I). One experiment has been conducted at the same time and with identical experimental conditions for the mutations T241P, R250P, D260V, T270I , as well as for Y232C, L224A (S-5), T235A (S-5), and G238A (S-6), therefore with the same Fas control, for both HIL-6 and HIL-6+3C conditions.

Supporting Information S-3

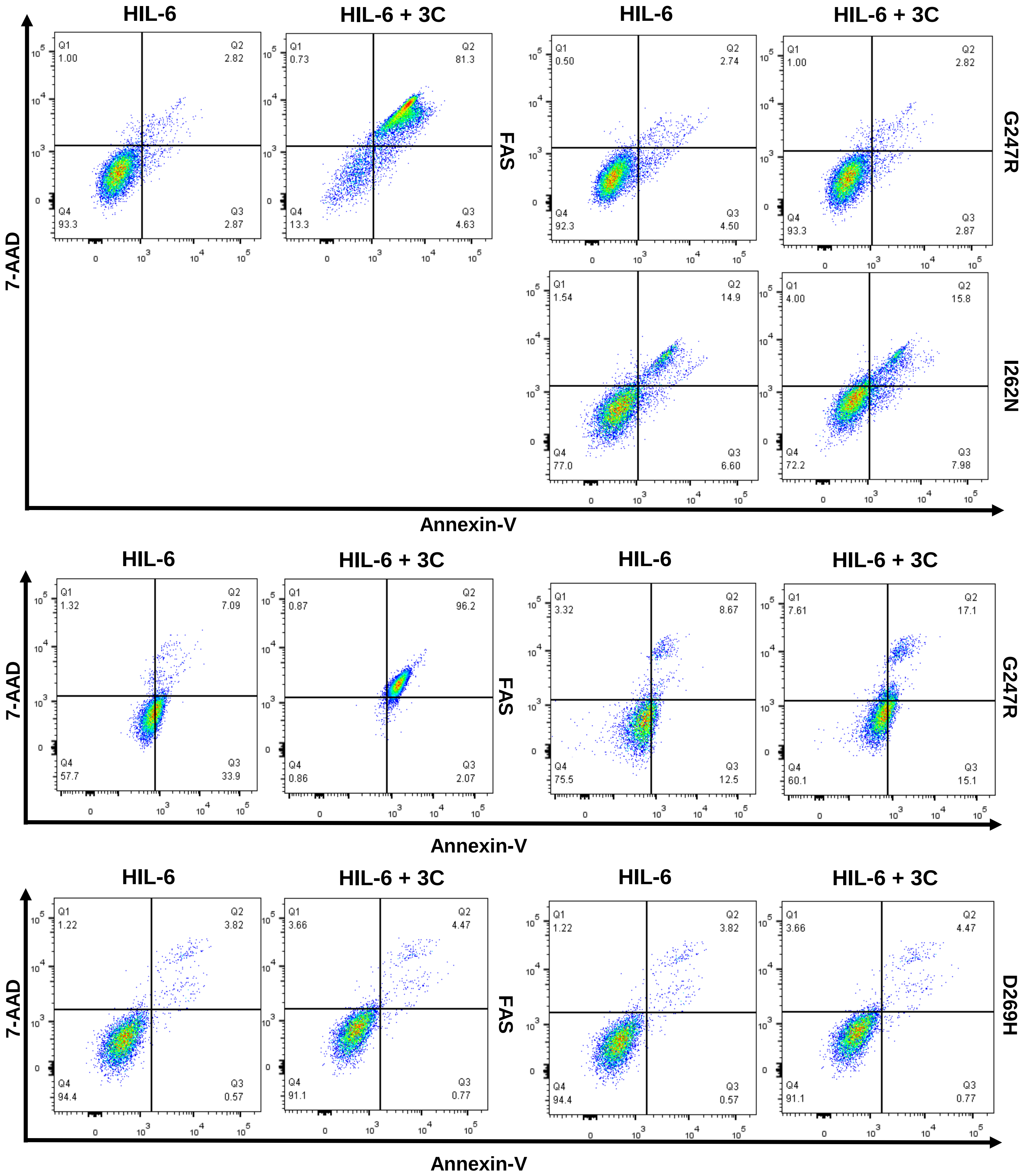

S-3: Flow-cytometry plots of apoptosis progression in Ba/F3-gp130 expressing C_{VHH}Fas wild-type and uncharacterized, LOF SNPs (G247R, G253V, I262N, D269H). One experiment has been conducted at the same time and with identical experimental conditions for the mutations G247R, I262N, E289D (S-4), Y291D (S-6), S230R (S-7), I233V (S-7), and L315F (S-7), as well as for G253V, N326H (S-4), and K251T (S-7), as well as for D269H and E272G (S-4), therefore with the same Fas control, for both HIL-6 and HIL-6+3C conditions.

Supporting Information S-4

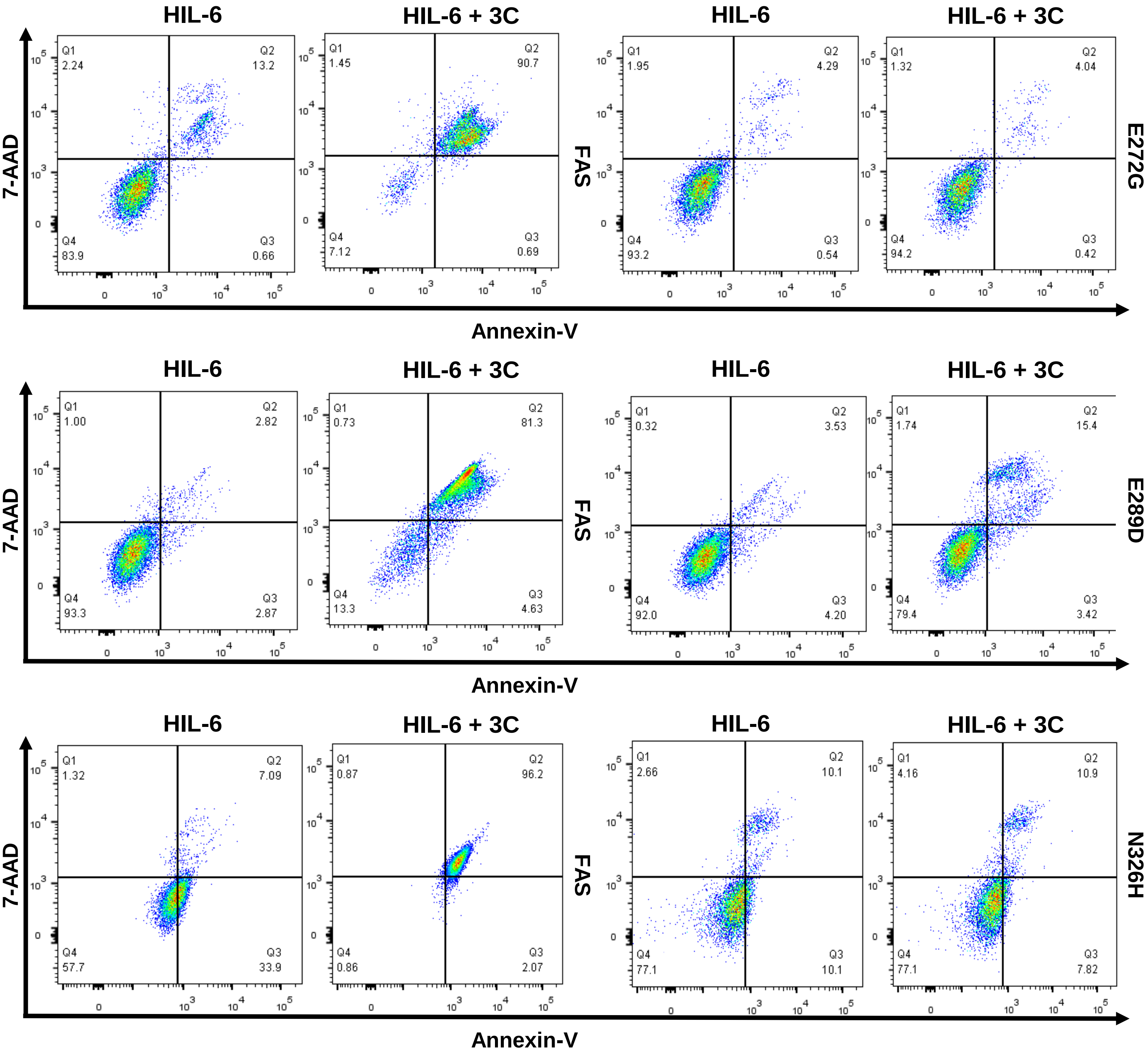

S-4: Flow-cytometry plots of apoptosis progression in Ba/F3-gp130 expressing C_{VHH}Fas wild-type and uncharacterized, LOF SNPs (E272G, E289D and N326H). One experiment has been conducted at the same time and with identical experimental conditions for the mutations E272G and D269H (S-3), as well as for E289D, G247R (S-3), I262N (S-3), Y291D (S-6), S230R (S-7), I233V (S-7), and L315F (S-7), as well as for N326H, G253V (S-3), and K251T (S-7), therefore with the same Fas control, for both HIL-6 and HIL-6+3C conditions.

Supporting Information S-5

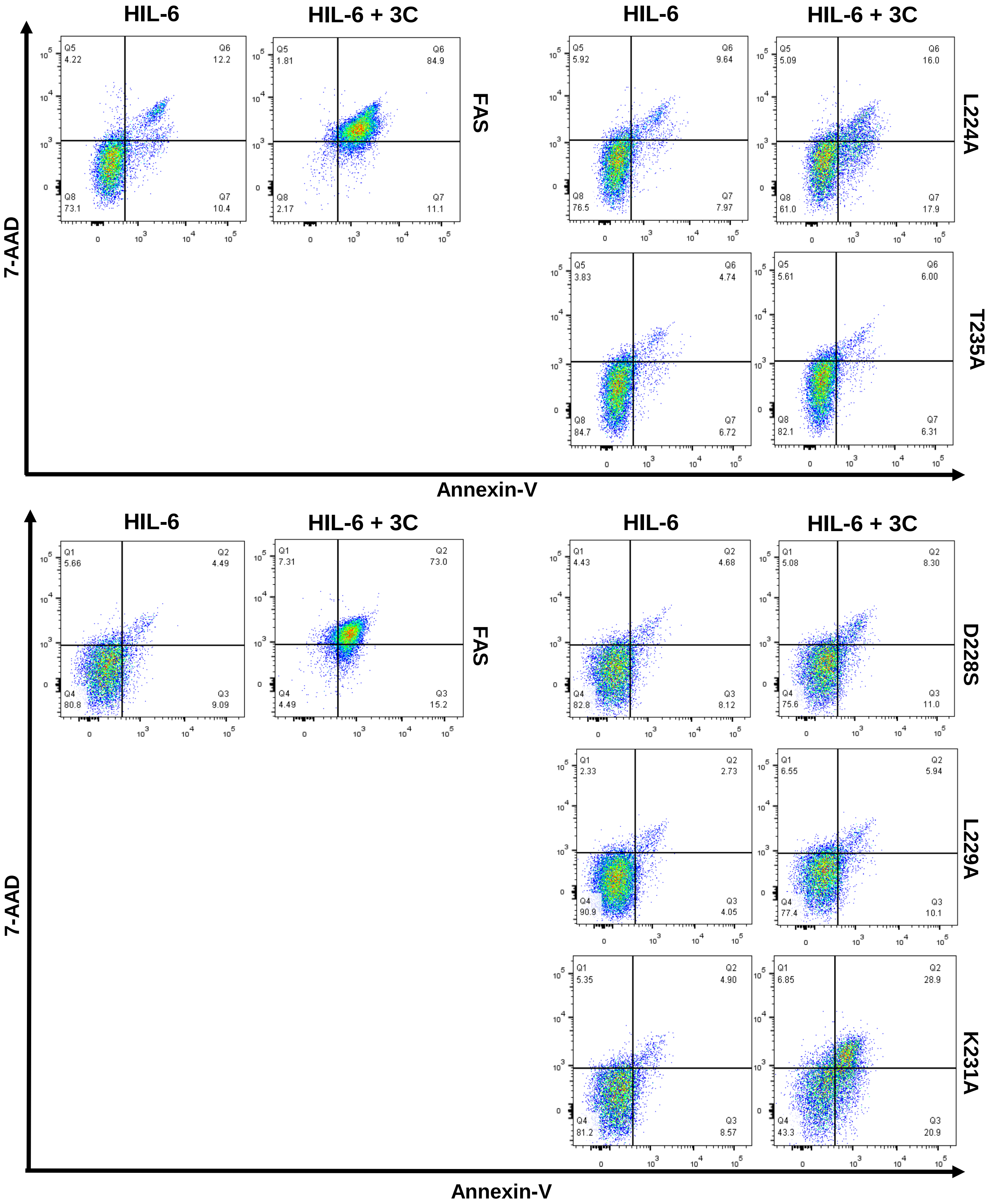

S-5: Flow-cytometry plots of apoptosis progression in Ba/F3-gp130 expressing C_{VHH}Fas wild-type and uncharacterized, LOF structure-based mutations (L224A, D228S, L229A, K231A, T235A). One experiment has been conducted at the same time and with identical experimental conditions for the mutations L224A, T235A, Y232C (S-2), and G238A (S-6), as well as for D228S, L229A, K231A, I236A (S-6), and L298A (S-6), therefore with the same Fas control, for both HIL-6 and HIL-6+3C conditions.

Supporting Information S-6

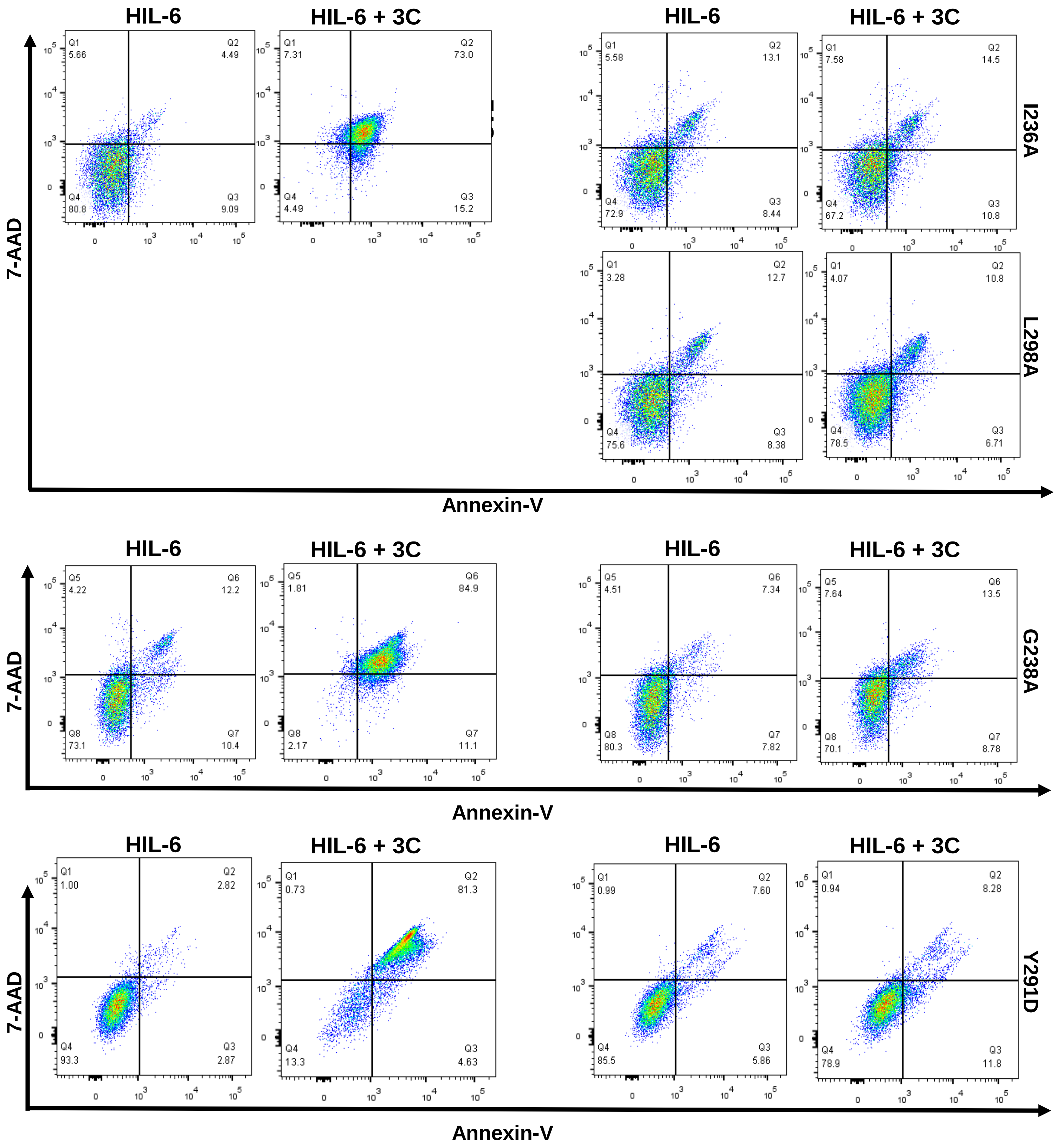

S-6: Flow-cytometry plots of apoptosis progression in Ba/F3-gp130 expressing C_{VHH}Fas wild-type and uncharacterized, LOF structure-based mutations (I236A, G238A, Y291D and L298A). One experiment has been conducted at the same time and with identical experimental conditions for the mutations I236A and L298A, D228S (S-5), L229A (S-5), K231A (S-5), as well as for G238A, Y232C (S-2), L224A (S-5), and T235A (S-5), as well as for Y291D, G247R (S-3), I262N (S-3), E289D (S-4), S230R (S-7), I233V (S-7), and L315F (S-7), therefore with the same Fas control, for both HIL-6 and HIL-6+3C conditions.

Supporting Information S-7

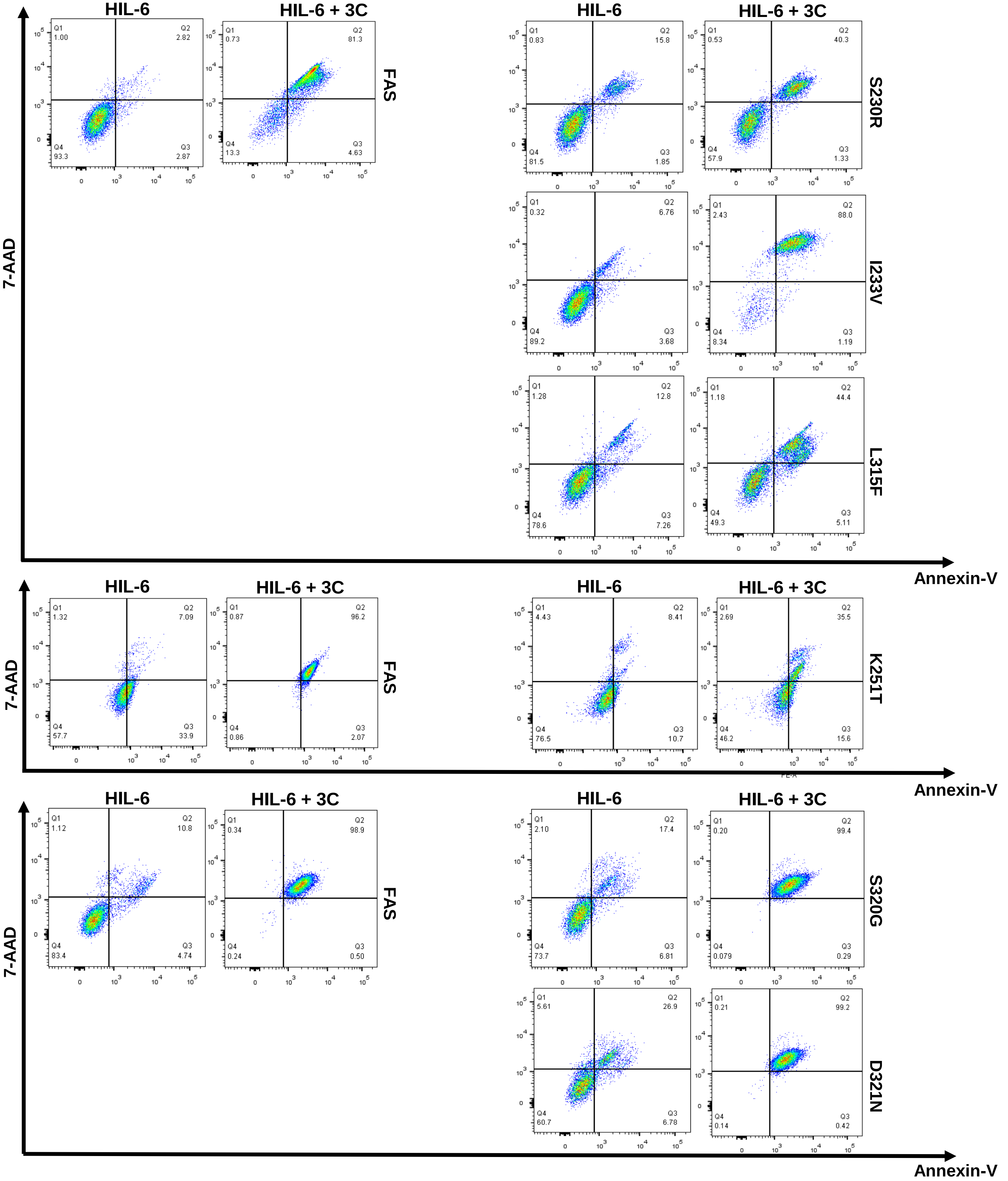

S-7: Flow-cytometry plots of apoptosis progression in Ba/F3-gp130 expressing C_{VHH}Fas wild-type and uncharacterized, active SNPs (S230R, I233V, K251T, L315F, S320G and D321N). One experiment has been conducted at the same time and with identical experimental conditions for the mutations S230R, I233V, and L315F, G247R (S-3), I262N (S-3), E289D (S-4), Y291D (S-6), as well as for K251T, G253V (S-3), N326H (S-4), as well as for S320G and D321N, therefore with the same Fas control, for both HIL-6 and HIL-6+3C conditions.

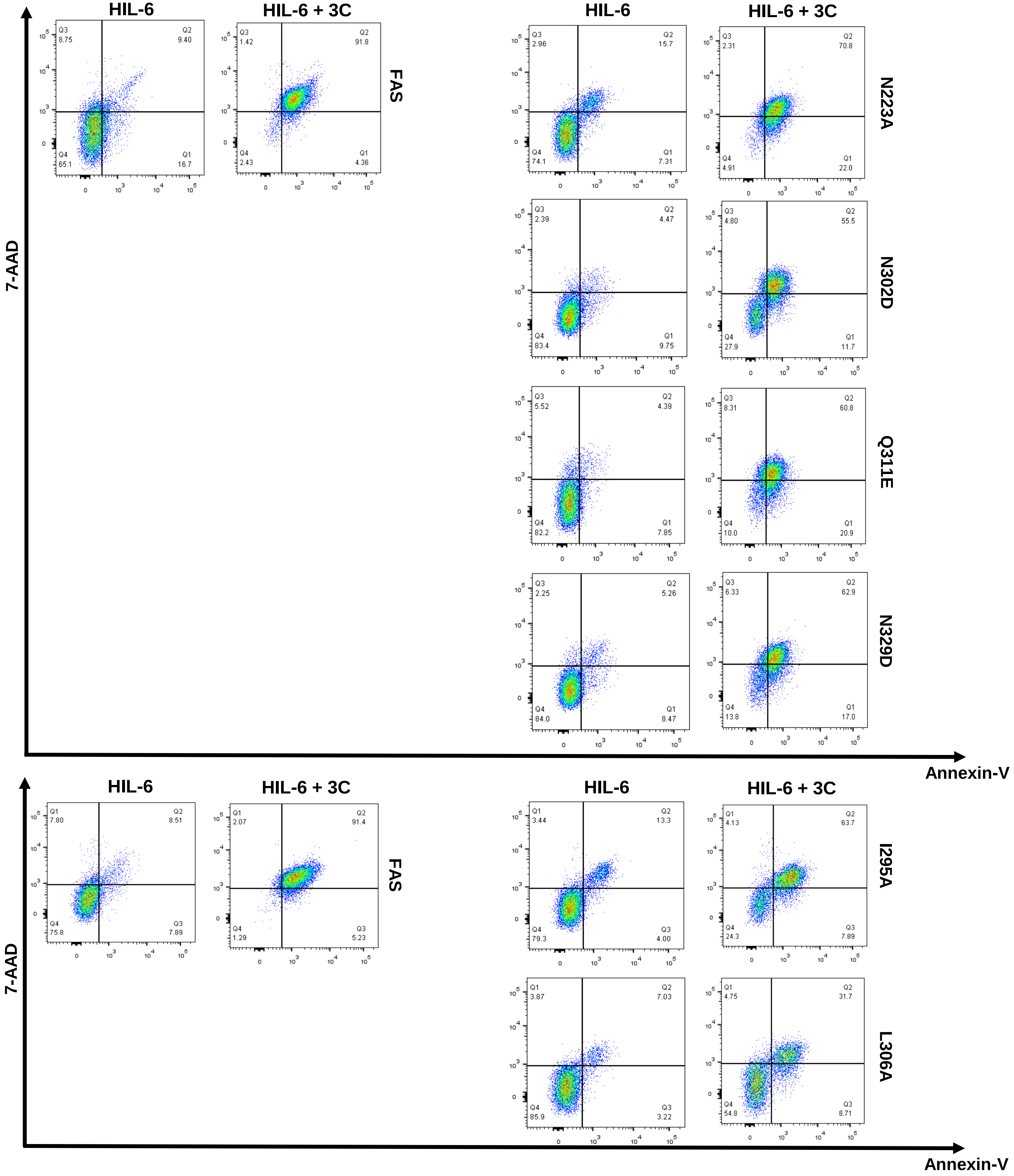

S-8: Flow-cytometry plots of apoptosis progression in Ba/F3-gp130 expressing C_{VHH}Fas wild-type and uncharacterized, active structure-based mutations (N223A, I295A, N302D, L306A, Q311E, N329D). One experiment has been conducted at the same time and with identical experimental conditions for the mutations N223A, N302D, Q311E, N329D, as well as for I295A, and L306A, therefore with the same Fas control, for both HIL-6 and HIL-6+3C conditions.

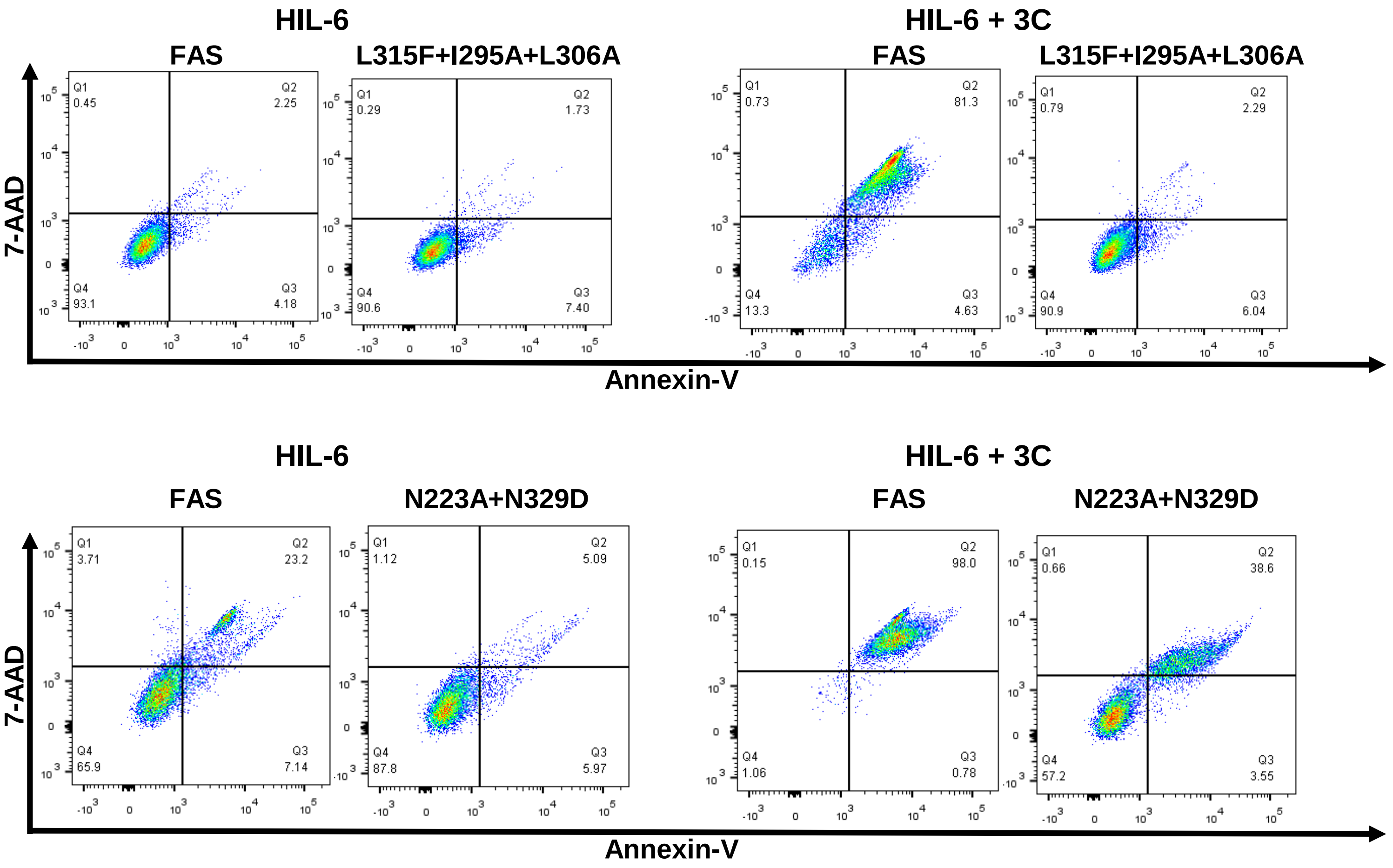

S-9: Flow-cytometry plots of apoptosis progression in Ba/F3-gp130 expressing C_{VHH}Fas wild-type and combination of L315F, I295A, L306A and N223A+N329D.

Supporting Information S-10

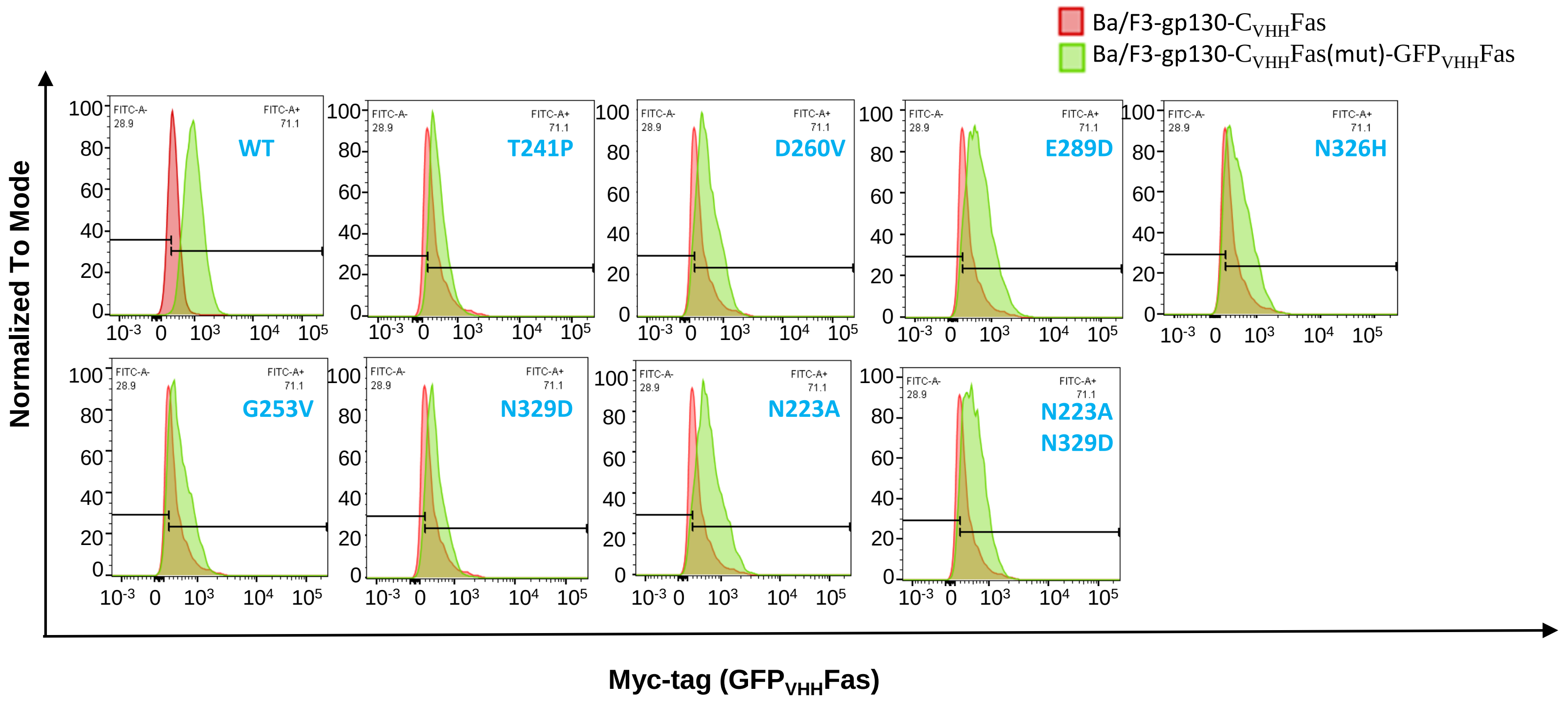

S-1: Cell surface expression of Ba/F3-gp130-C$_{VHH}$Fas as negative control (red) and Ba/F3-gp130-C$_{VHH}$Fas-GFP$_{VHH}$Fas (green) by specific detection of surface Myc-tag through flow-cytometry.

Supporting Information S-11

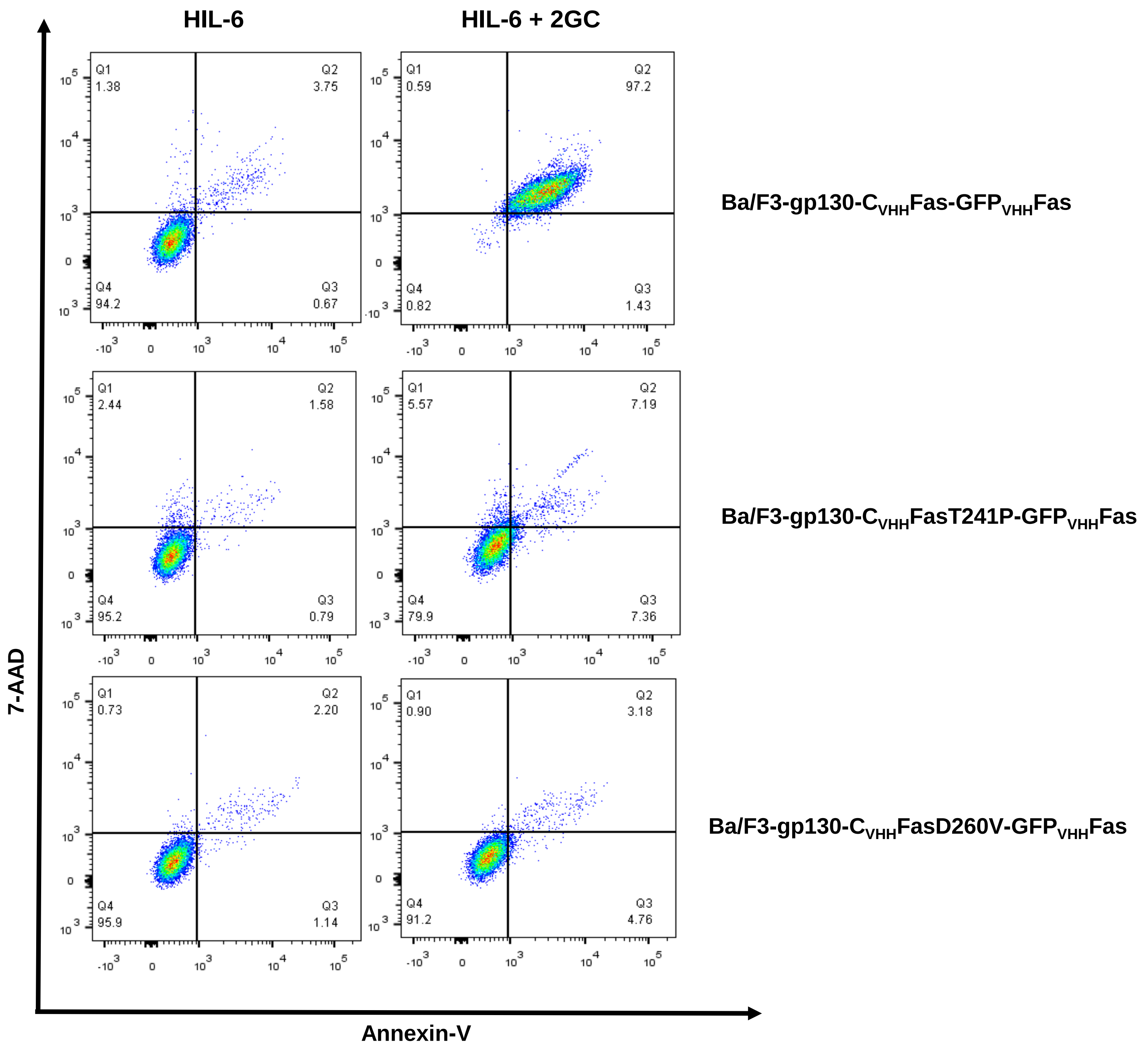

S-11: Flow-cytometry plots of apoptosis progression in Ba/F3-gp130 expressing wild-type and mutated C$_{VHH}$Fas and wild type GFP$_{VHH}$Fas. One experiment has been conducted at the same time and with identical experimental conditions for the mutations T241P, D260V, and for E289D, N326H, G253V (S-12), and for N329D, N223A, N329D+N223A (S-13), therefore with the same Fas control, for both HIL-6 and HIL-6+2GC conditions.

Supporting Information S-12

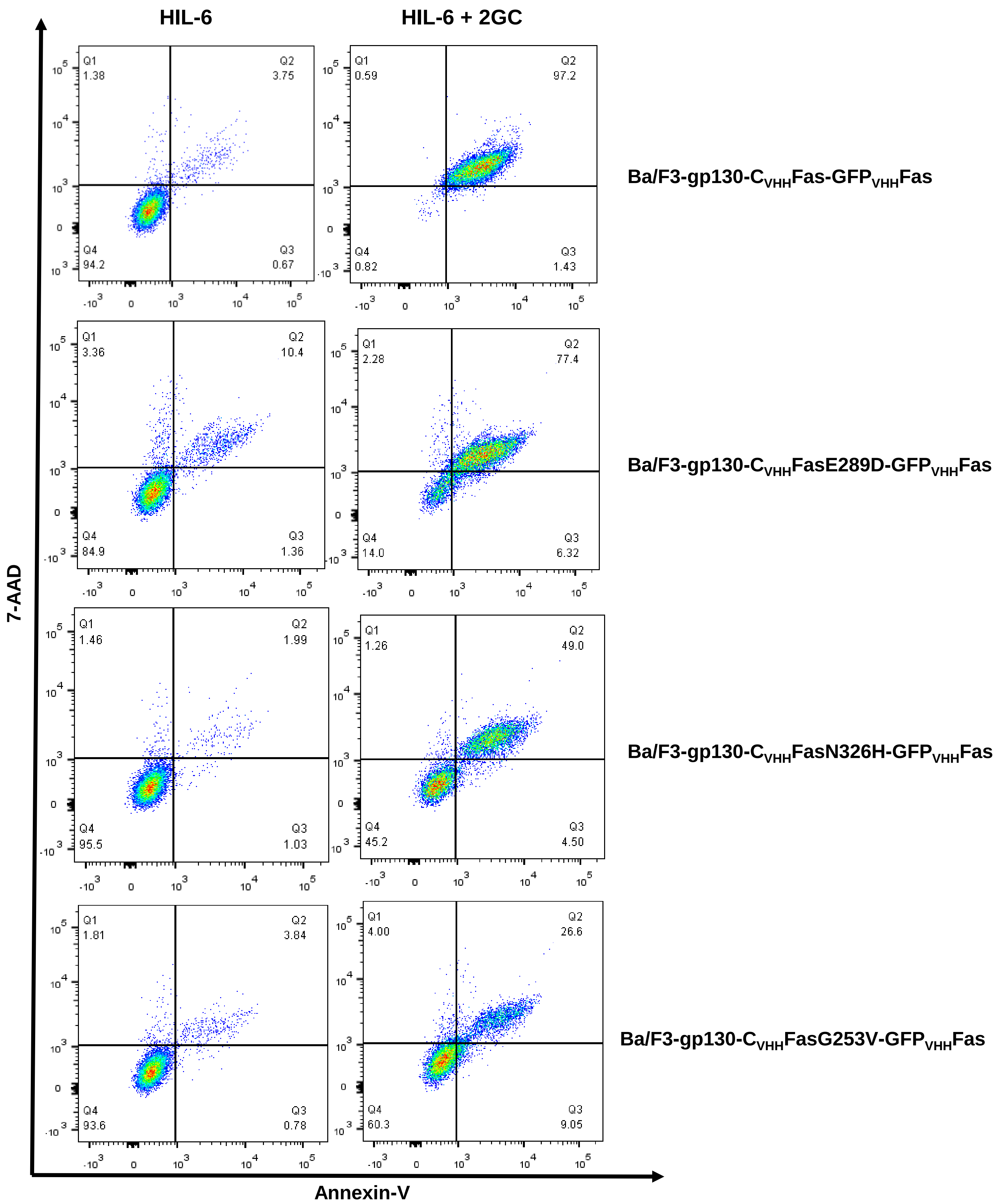

S-12: Flow-cytometry plots of apoptosis progression in Ba/F3-gp130 expressing wild-type and mutated C$_{VHH}$Fas and wild type GFP$_{VHH}$Fas. One experiment has been conducted at the same time and with identical experimental conditions for the mutations E289D, N326H, G253V, and for T241P, D260V (S-11), and for N329D, N223A, N329D+N223A (S-13), therefore with the same Fas control, for both HIL-6 and HIL-6+2GC conditions.

Supporting Information S-13

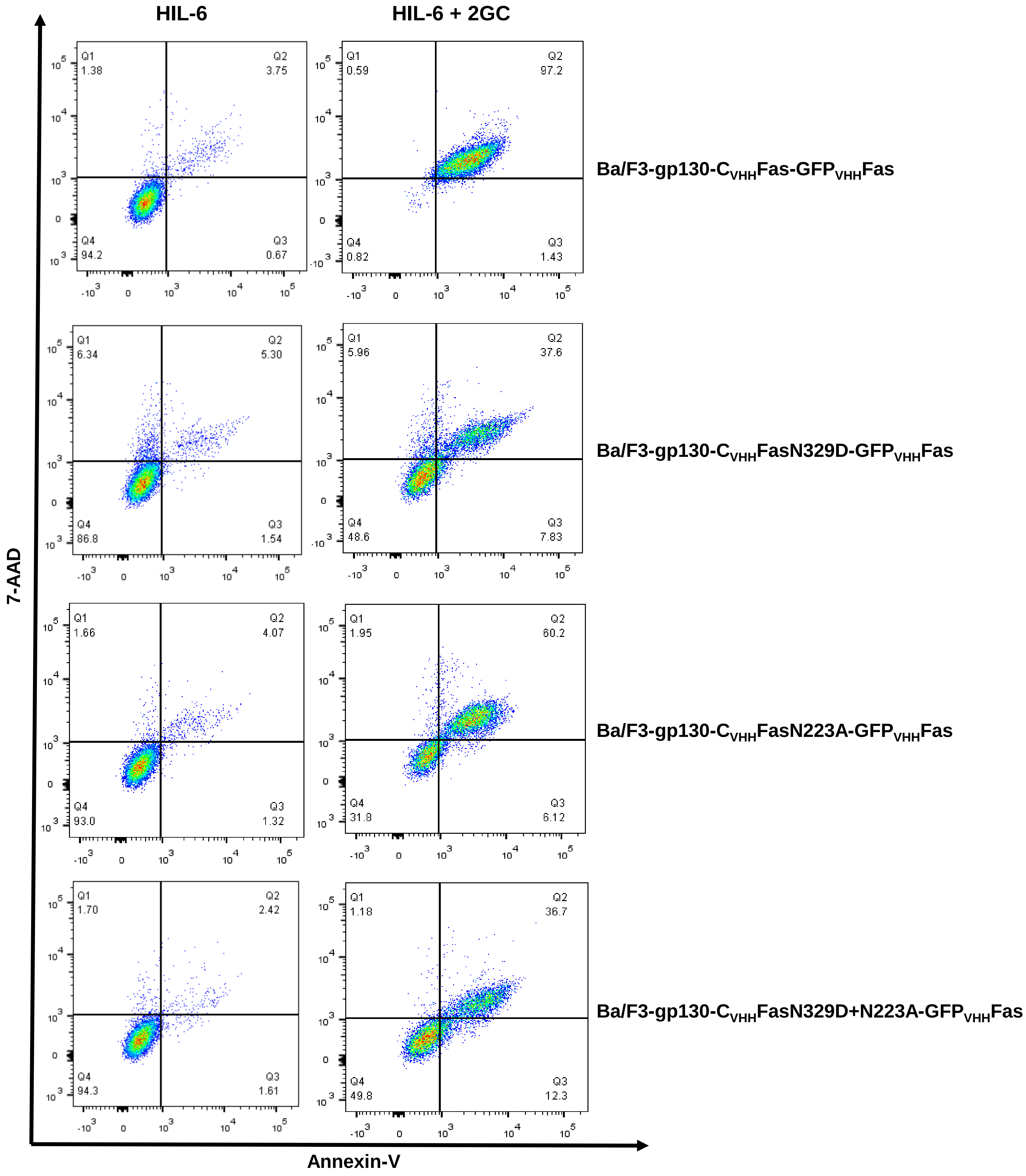

S-13: Flow-cytometry plots of apoptosis progression in Ba/F3-gp130 expressing wild-type and mutated C$_{VHH}$Fas and wild type GFP$_{VHH}$Fas. One experiment has been conducted at the same time and with identical experimental conditions for the mutations N329D, N223A, N329D+N223A, and for T241P, D260V (S-11), and for E289D, N326H, G253V (S-12), therefore with the same Fas control, for both HIL-6 and HIL-6+2GC conditions.
